# Arm and head domain in highly conserved lipoprotein modification enzyme Lgt determine functional diversity among bacterial pathogens

**DOI:** 10.1128/mbio.00600-26

**Published:** 2026-05-13

**Authors:** Simon Legood, Ana Oliveira Paiva, Najwa Taib, Tristan Ruffiot, Simonetta Gribaldo, Ivo Gomperts Boneca, Nienke Buddelmeijer

**Affiliations:** 1 Institut Pasteur, Université Paris Cité, Unité Biologie et Génétique de la Paroi Bactérienne, UMR6047 CNRS, INSERM U1306 27058, Paris, France; 2 Institut Pasteur, Université Paris Cité, Unité Biologie Évolutive de la Cellule Microbienne 555089, Paris, France; National University of Singapore, Singapore, Singapore

**Keywords:** lipoproteins, Lgt, pathogens, functional diversity, structural conservation

## Abstract

The post-translational lipoprotein modification pathway is conserved in bacteria, in which prolipoprotein phosphatidylglycerol diacylglyceryl transferase (Lgt) catalyzes the first and committed step. Due to its essentiality for cell viability in Proteobacteria, its membrane localization, and relative accessibility, Lgt is proposed as a promising target for the development of novel antibiotics. To answer the question of the degree of conservation between Lgt homologs of WHO-listed pathogenic species, we performed evolutionary, structural, and functional analyses. Our data show that Lgt is present in all bacteria and absent from archaea. AlphaFold structural models are similar to the X-ray structure of Lgt from *E. coli* with most variability and less conserved residues in the arm and head domains between Lgt homologs. Lgt of diderm bacteria, but not of monoderm bacteria, restores growth and viability of an Lgt depletion strain in *E. coli*. Sequence alignments and site-directed mutagenesis demonstrate that unique conserved residues on arm-2 together with histidine 103 and the periplasmic head domain, determine protein substrate specificity. This large-scale analysis led to the definition of an Lgt motif and an alternative catalytic mechanism. Our results highlight similarities in catalytic mechanism and differences in substrate specificity between Lgt homologs from pathogenic species, with an impact on strategies to develop narrow-spectrum antibiotics targeting Lgt.

**IMPORTANCE:**

Antimicrobial resistance is a major threat to public health, for which the identification of novel targets and the development of new therapies are urgently needed. The bacterial lipoprotein modification pathway is promising for the exploration of new antibiotics since it is unique to bacteria, essential for bacterial viability and virulence, and accessible to drugs due to the exposed domains of the modification enzymes. In this study, we explored large-scale sequence analysis, structural modeling, and functional assays of the first enzyme in the pathway. Our findings show that the enzyme is highly conserved across distant phyla, that homologous enzymes have similar structures and contain a signature motif composed of invariant essential residues, but that functional conservation divides monoderm and diderm pathogenic bacteria. This correlates with structural variation and differences in substrate specificity, illustrating the potential for the development of narrow-spectrum antibiotics targeting the lipoprotein modification pathway.

## INTRODUCTION

The bacterial cell envelope fulfills an important function in bacterial physiology by maintaining cell shape and protecting against external threats and environmental stress (1). It also plays an important role in pathogenic species through interactions between its virulence factors and host cells, leading to signaling of the innate immune response (2). Lipoproteins are important components of the cell envelope, where they are anchored into phospholipid membranes via their lipid N-terminus (3). In the canonical lipoprotein modification pathway, well characterized in *Escherichia coli*, preprolipoprotein phosphatidylglycerol diacylglyceryl transferase (Lgt) is the first enzyme that adds a diacylglyceryl (DAG) moiety from phosphatidylglycerol (PG) onto the invariant cysteine, resulting in a thioether-linked prolipoprotein (4). Signal peptidase II (Lsp) cleaves the signal peptide (5), followed by apolipoprotein N-acyltransferase (Lnt), which transfers a fatty acid from *sn*-1 of phosphatidylethanolamine, resulting in a triacylated mature lipoprotein (6). Although the first two steps are thought to be consistent in all bacteria, variation occurs in the third step of modification. In *Bacteroides*, N-acylation is catalyzed by Lnb (7), which is structurally and catalytically different from Lnt of *E. coli* (8). Monoderm bacteria also perform N-acylation of lipoproteins (9) by the combined action of two enzymes, LnsA and LnsB (10). These bacteria can also transfer fatty acids from S-diacylglyceryl cysteine to the free α-amine group of the same residue via lipoprotein intermolecular transferase (Lit) (11, 12). In monoderm bacteria, where lipoprotein modification is not essential for viability, the degree of lipidation results in attenuated or enhanced virulence depending on the species (13). Surface-exposed lipoproteins in diderm bacteria are potent virulence factors and are developed (14, 15) or explored as vaccine candidates (16).

The lipoprotein modification pathway is considered an excellent target for the development of novel antibacterials (16, 17). Indeed, Lsp inhibitors, globomycin (18) and myxovirescin (19), have been known for many years, and the structure of the enzyme-inhibitor complexes has been solved (20). More recently, two inhibitors of Lgt were identified by selecting cyclic peptides that bind Lgt (21) and in a high-throughput screen with small molecules that inhibit *in vitro* growth of *E. coli* (22). Both cyclic peptide G2428 and inhibitor MAC-0452936 inhibit Lgt activity *in vitro*. The X-ray crystal structure of Lgt from *E. coli* depicts a compact integral membrane enzyme composed of seven transmembrane domains (body), two short β-sheets, and one α-helix (arms) that align on top of the cytoplasmic membrane and a periplasmic domain (head) (23). A central cavity contains essential residues, where the DAG transfer reaction is predicted to take place. A catalytic mechanism is proposed with a central role for histidine 103, together with residues in the central cavity that interact with the polar headgroup of PG (24, 25). Binding of preprolipoprotein is proposed to induce a conformational change in Lgt such that histidine 103 moves close to the phospholipid and the protein substrate and acts as a catalytic base to activate cysteine in preprolipoprotein for a nucleophilic attack on C3 of PG (23, 25).

Here, we asked to what degree Lgt is conserved on an evolutionary, structural, and functional level in bacteria, particularly in pathogenic species that pose a serious threat to human health. Our results show that specific amino acids in a structurally highly conserved protein determine functional variability between Lgt homologs, suggesting that Lgt is suitable for the development of narrow-spectrum antibiotics.

## RESULTS

### Primary sequence and phylogenetic analysis show high conservation of Lgt proteins across distant phyla

**Fig 1:**
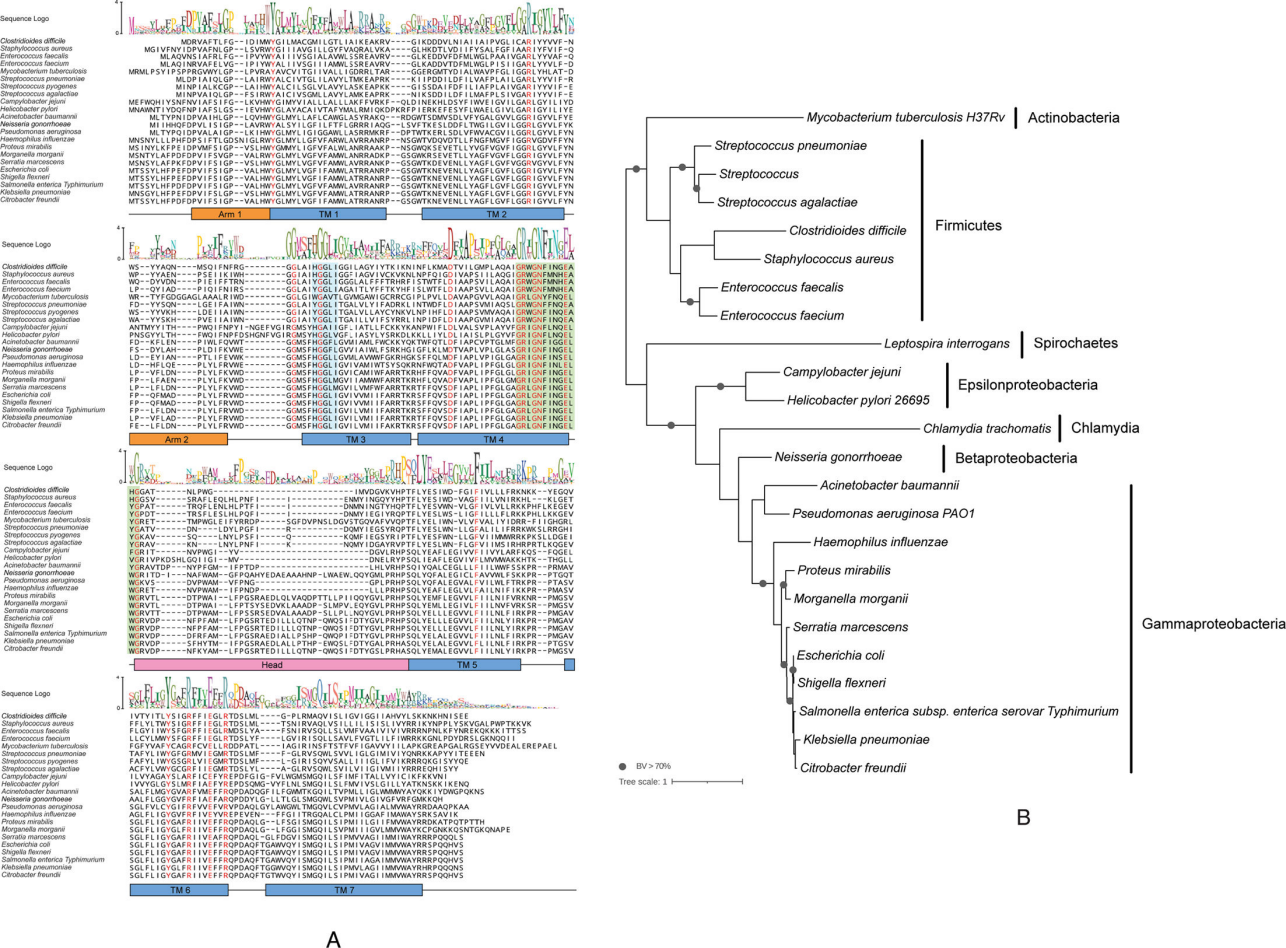
Sequence alignment and phylogenetic tree of Lgt proteins. (**A**) Twenty-two Lgt proteins were selected from bacterial pathogens; seven species belong to Firmicutes and 15 to Proteobacteria. The degree of amino acid conservation is indicated in sequence logo, with 16 fully conserved residues shown in red. The conserved motif H_103_GGL (blue), containing active site residue H103 (W/Y), and the Lgt signature motif G_142_R_143_LGN_146_FINGE_151_LWR (green) are highlighted. Transmembrane domains TM1-TM7 (blue), periplasmic head domain (corresponding to β4-β5-α3-α4-β6 in the X-ray structure) (pink), and arm-1 (β2-β3) and arm-2 (α1-η2) (orange) are shown (23). The Lgt sequence of *M. tuberculosis* is truncated at the C-terminus at position 303 to eliminate the non-homologous extension (see Fig. 2B). (**B**) The maximun-likelihood tree was inferred from an alignment of 24 sequences, including Lgt from *Leptospira interrogans* and *Clamydia trachomatis* (Fig. S1), and 222 amino acid positions, using the model LG+F+G4. The scale bar represents the average number of substitutions per site. BV, bootstrap values.

**Fig 2:**
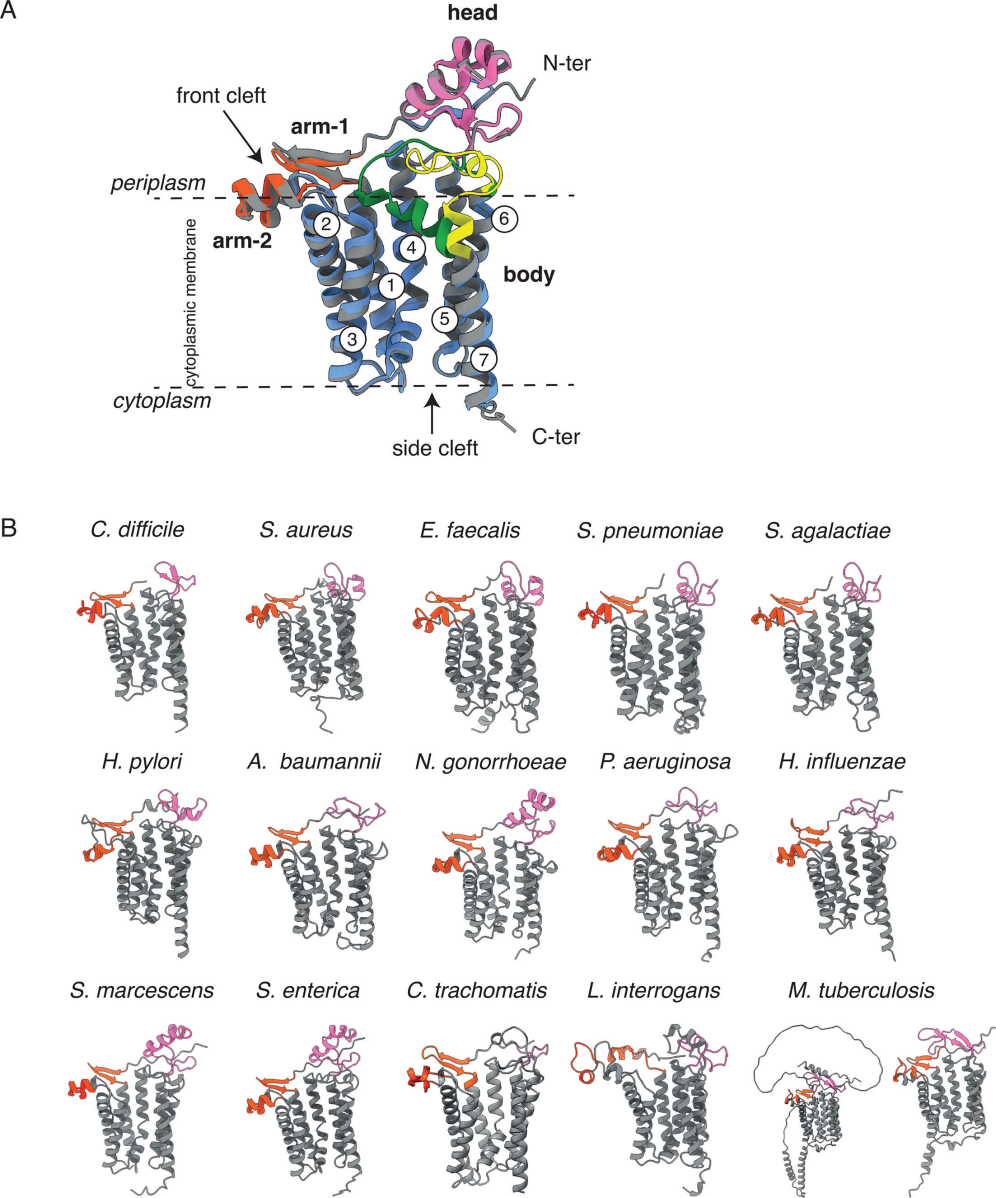
*E. coli* Lgt X-ray structure and AlphaFold 2 models for Lgt from selected pathogenic species. (**A**) Comparison of X-ray structure PDB 5azc (blue) (23) and AlphaFold (AF2) structure model (gray) of *E. coli* Lgt. TMs are numbered according to Fig. 1A. Differences in the conformation of TM7 and the loop connecting TM6 and 7 between X-ray structure and AF2 model are indicated in green and yellow, respectively. Entry of substrate(s) is predicted to occur at the front cleft formed by arm-1 and arm-2 (shown in orange in 5azc), and exit of diacylglyceryl prolipoprotein is predicted to take place via the side cleft (25). The head domain (pink in 5azc) is exposed to the periplasm. (**B**) AF2 structural models of 15 Lgt proteins of pathogenic species, as shown in Fig. 1 and Fig. S1. Arm domains are shown in orange and head domains in pink. Full-length Lgt from *M. tuberculosis* is shown on the left, and the truncated version (from residue 303) is shown on the right.

We searched Lgt homologs in 22 bacterial species belonging to the Proteobacteria and Firmicutes phyla, listed as priority pathogens (26–29), using the SyntTax database (30). The retrieved sequences were aligned, and a maximum likelihood phylogeny was inferred to obtain insight into the degree of evolutionary conservation. Overall, the multiple sequence alignment (MSA) showed poor conservation in the arms and periplasmic head domains (Fig. 1A). It revealed 16 strictly conserved residues, including amino acids postulated to be involved in catalysis (23, 25). Thirteen residues were previously shown essential for viability (23, 24, 31, 32), among which histidine 103 found in Proteobacteria but not in Firmicutes and Actinobacteria, where Y and W residues replace H103, respectively. Lgt of *Mycobacterium tuberculosis* has a large C-terminal extension that has no homology with known proteins (Fig. 2B). We included Lgt from *Chlamydia trachomatis* (Chlamydia) and *Leptospira interrogans* (Spirochaetes) as representative species that map between Firmicutes and Proteobacteria on a phylogenetic tree (Fig. 1B). The sequence alignment shows poor conservation of essential residues, in particular for *L. interrogans* Lgt, compared to *E. coli* (Fig. S1).

The Lgt phylogenetic tree has two distinct clades: the first containing Lgt sequences from the phyla Firmicutes and Actinobacteri, and the second from Proteobacteria, Chlamydia, and Spirochaetes (Fig. 1B). The monophyly of these five phyla suggests that no recent horizontal transfer of the *lgt* gene occurred among their members.

### Lgt structure is highly conserved with main variations in the periplasmic (head) domain

Two X-ray crystal structures of Lgt from *E. coli* have been previously solved: form-1 (PDB 5azb) with palmitate and β-octylglucoside, and form-2 (PDB 5azc), with PG and DAG, referred to as the active form of the enzyme (23). The essential and conserved residues reside on top of TM3 and TM4 and in TM5 and TM6, whereas the exposed periplasmic (head) domain is the least conserved (Fig. 1A and 2A).

Structural conservation of Lgt homologs was assessed using the structure prediction program AlphaFold2 (AF2) (33). Confidence metrics for Lgt of *E. coli* indicate high confidence by pIDDT and low prediction error as defined by PAE; however, for the region at the periplasmic half of TM7 and the loop between TM6 and TM7 (L6-7) (around residue 250; *E. coli* numbering), low pIDDT and high PAE scores were observed suggesting flexibility in this region (Fig. S2A). The X-ray structure has an extended α-helix (Fig. 2A; shown in green) that arcs below the membrane plain, whereas the predicted AF2 model has a shortened α-helix and unstructured loop (Fig. 2A; shown in yellow) extending upward through the membrane.

From the 24 selected Lgt proteins, we chose 15 based on pathogen priority profile and degree of antibiotic resistance and modeled them using AF2 (Fig. 2B). Due to the extended C-terminus of Lgt of *M. tuberculosis*, low confidence and high prediction error were calculated (Fig. S2B). Main differences among models were observed in L6-7, in the loop between arm-2 and TM3 (around residue 100), and at the N- and C-terminus (Fig. 2B; Fig. S2A through D). The most striking difference in structure, consistent with poor primary sequence conservation, is found in the periplasmic (head) domain (Fig. 2B). Lgt of Enterobacteriales, Spirochaetes, and β-Proteobacteria have a similarly large head domain, whereas Lgt of more distant related Proteobacteria, Firmicutes, and Chlamydia have smaller less prominent head domains that are predicted to be located closer to the membrane interface (Fig. 1A and 2B; Fig. S1). These results suggest that, although Lgt proteins are highly conserved and catalyze the same diacylglyceryl transfer reaction, they may differ in enzymatic activity and/or substrate specificity.

### Lgt of diderm bacteria are functional in *E. coli*

We sought to determine the degree of functional conservation of Lgt from different species by employing complementation studies using the Lgt depletion strain of *E. coli*, where the endogenous *lgt* gene is replaced by a kanamycin resistance cassette and *lgt* is expressed under control of an arabinose inducible promoter at a distinct locus (21). Genes encoding Lgt of species representing different branches of the phylogenetic tree were expressed from a P*_lac_* promoter on a low-copy expression vector by induction with IPTG (32, 34) (Table S1).

**Fig 3:**
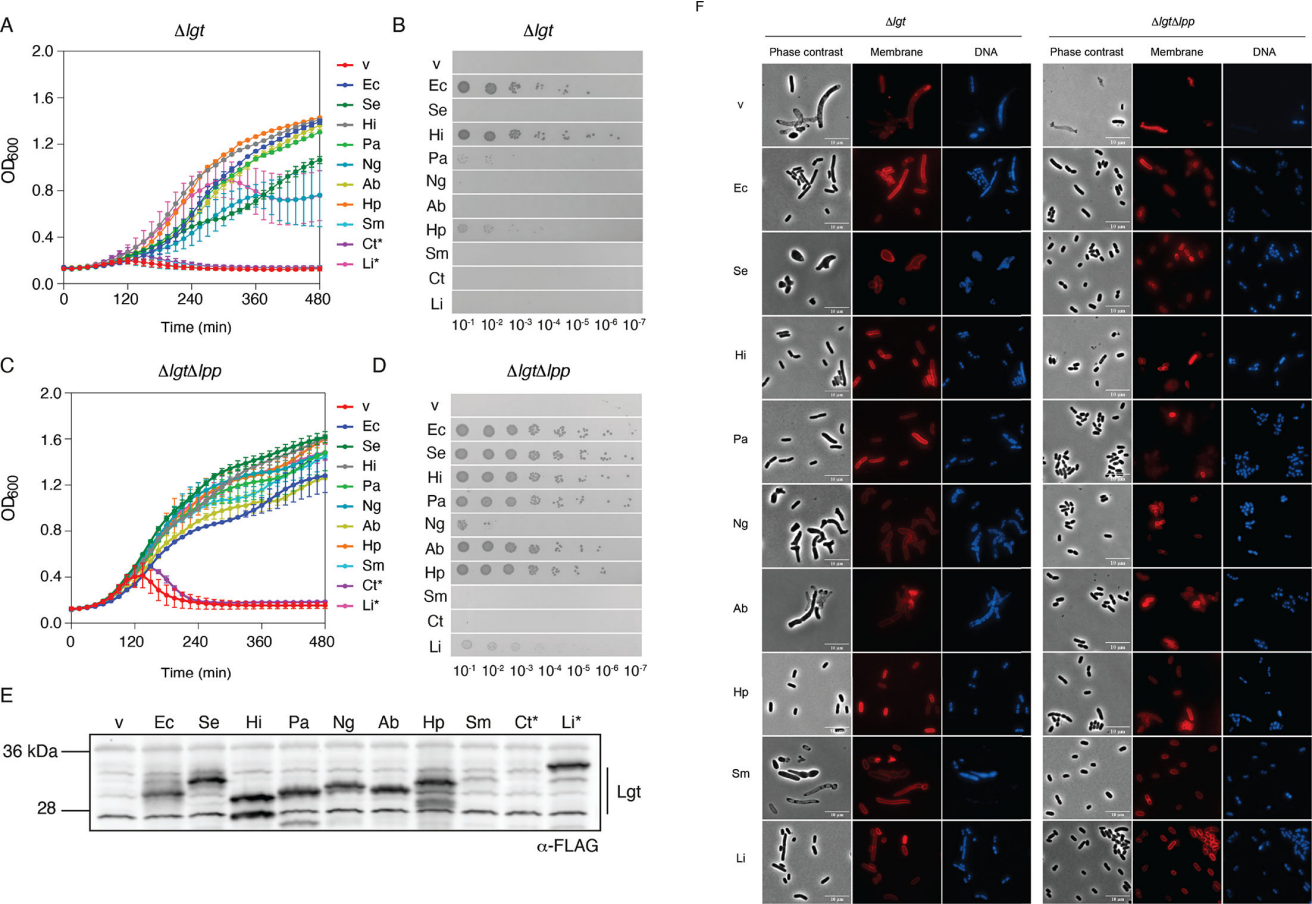
Lgt from diderm bacteria complement an *E. coli* Lgt depletion strain in the absence of major lipoprotein Lpp. (**A**) Growth of Δ*lgt* (SLEC67) (34) carrying low-copy plasmid pAM238 encoding Lgt homologs in 0.2% D-glucose and 5 mM IPTG. After initial 2-hour depletion of Lgt through growth in the absence of sugar, cultures were grown in 96-well plates in a TECAN spectrophotometer, and OD_600_ was recorded at regular intervals. Assays were completed at least in duplicates; error bars indicate standard deviation from the mean. v, empty vector; Ec, *E. coli*; Se, *S. enterica* serovar Thyphimurium; Hi: *H. influenza*; Pa, *P. aeruginosa*; Ng, *N. gonorrhoeae*; Ab, *A. baumannii*; Hp, *H. pylori*; Sm, *S. marcescens*; Ct*, codon-optimized *lgt* sequence from *C. trachomatis*; Li*, codon-optimized *lgt* sequence from *L. interrogans*. (**B**) Spot dilution assay of strains grown in the presence of 0.2% D-glucose and 5 mM IPTG corresponding to *T*_0_ of the growth curve. Assays were completed in duplicate. (**C**) Growth of Δ*lgt*Δ*lpp* (SLEC68) (34) carrying low-copy plasmid pAM238 encoding Lgt homologs under the same conditions as in panel **A**. (**D**) Spot dilution assay of SLEC68 derived strains grown in the presence of 0.2% D-glucose and 5 mM IPTG corresponding to *T*_0_ of the growth curve as shown in panel **C**. (**E**) Detection of Lgt-FLAG_3_ proteins with anti-Flag antibodies upon expression of *lgt-flag_3_* from low-copy plasmid pAM238 in *E. coli* MG1655^Q^ by addition of 5 mM IPTG. Total cell lysates corresponding to 0.1 OD_600_ units were loaded to each lane. (**F**) Microscopy images of fixed cells corresponding to *T*_270_ of the growth curves. C. *trachomatis* was not included because Lgt protein could not be detected on Western blot. Membranes were stained with lipophilic dye FM4-64X (red), and nucleoids were stained with Hoechst 2333 (blue). Scale bar: 10 μm.

We performed growth kinetics and cell viability analyses to determine the capacity of Lgt homologs to restore growth in liquid medium and to form colonies, respectively. When chromosomal *lgt* expression was repressed by growth in D-glucose and P*_lac_* constructs carrying homologous *lgt* induced with IPTG, growth and viability were not restored in the presence of the empty vector (v) (Fig. 3A). When *lgt* of *E. coli*, *Haemophilus influenzae*, *Pseudomonas aeruginosa*, *Acinetobacter baumannii*, and *Helicobacter pylori* were expressed in Δ*lgt*, similar growth curves were observed (Fig. 3A). Growth was delayed with Lgt from *Salmonella enterica* serovar Thyphimurium and *Neisseria gonorrhoeae*, and no growth was observed with Lgt from *Serratia marcescens* (Fig. 3A). Colonies formed with Lgt of *E. coli* and *H. influenzae*, and in lower numbers with Lgt of *P. aeruginosa* and *H. pylori* (Fig. 3B). No colonies were observed with Lgt from *S. enterica* serovar Thyphimurium, *N. gonorrhoeae*, *A. baumannii*, and *S. marcescens* (Fig. 3B).

All Lgt homologs were produced in wild-type *E. coli* (Fig. 3E), and while protein quantity is variable, similar amounts were observed, except for Lgt of *S. marcescens* which is less produced (Fig. 3E).

Cell morphology was strongly affected in Δ*lgt* cells producing Lgt of *S. enterica* serovar Thyphimurium, *N. gonorrhoeae*, *A. baumannii*, and *S. marcescens*; large, branched, and lysed cells were observed, but complementing Lgt homologs of *H. influenzae*, *P. aeruginosa*, and *H. pylori* led to normal cell morphology (Fig. 3F). Strikingly, while Lgt from *S. enterica* serovar Thyphimurium is a close homolog of *E. coli,* it did not restore morphology and viability of Δ*lgt*. Lgt protein levels were similar for *E. coli* and *S. enterica* serovar Thyphimurium. Similarly, an aberrant cell morphology was observed with *S. marcescens* Lgt, another closely related species (Fig. 3F), and colonies were not formed on plates (Fig. 3D). Protein levels of *S. marcescens* Lgt were lower than Lgt from *E. coli* and *S. enterica* serovar Thyphimurium.

We previously showed that lipoprotein Lpp is restricted to a subclade of γ-Proteobacteria (35), which, when mis-localized to the cytoplasmic membrane and covalently cross-linked to the peptidoglycan, leads to cell lysis (36). We demonstrated that Lgt is essential for growth and viability of *E. coli* in the absence of Lpp, highlighting the importance of other lipoproteins, including essential lipoproteins LolB, LptE, and BamD, for physiology (34). Due to the high abundance of Lpp, we reasoned that subtle differences in function between Lgt homologs could be observed in a Lgt depletion strain lacking Lpp (Δ*lgt*Δ*lpp*) (34). Restoration of growth, viability, and morphology of Δ*lgt*Δ*lpp* was observed with basal-level *E. coli lgt* expression (without induction with IPTG), demonstrating that low levels of Lgt are sufficient for complementation (Fig. S3).

Growth of all strains expressing proteobacterial *lgt* was fully restored in liquid media in the presence of IPTG (Fig. 3C), and cell morphology was restored in the absence of Lpp (Fig. 3F). These data suggest that some Lgt homologs are less efficient in modifying *E. coli* lipoproteins and are functional when substrate load is reduced through elimination of Lpp.

Lgt from diderm bacteria that branch between Firmicutes and Proteobacteria on the phylogenetic tree were analyzed for their capacity to complement the Lgt depletion mutant of *E. coli. L. interrogans* Lgt was produced in *E. coli* and restored growth of Δ*lgt* up to mid-log phase without a decrease in optical density after 300 min (Fig. 3A), but colonies were not formed (Fig. 3B). In Δ*lgt*Δ*lpp*, growth was similar to *E. coli* Lgt, and small colonies were observed (Fig. 3D). Cell morphology was similar in Δ*lgt* and Δ*lgt*Δ*lpp* backgrounds (Fig. 3F). No protein was detected for Lgt from *C. trachomatis* (Fig. 3E)*,* which correlates with a non-complementation phenotype in *E. coli* (Fig. 3A through D). Microscopic analysis was therefore not included for *C. trachomatis* Lgt.

### Lgt of monoderm bacteria are not functional in *E. coli*

**Fig 4:**
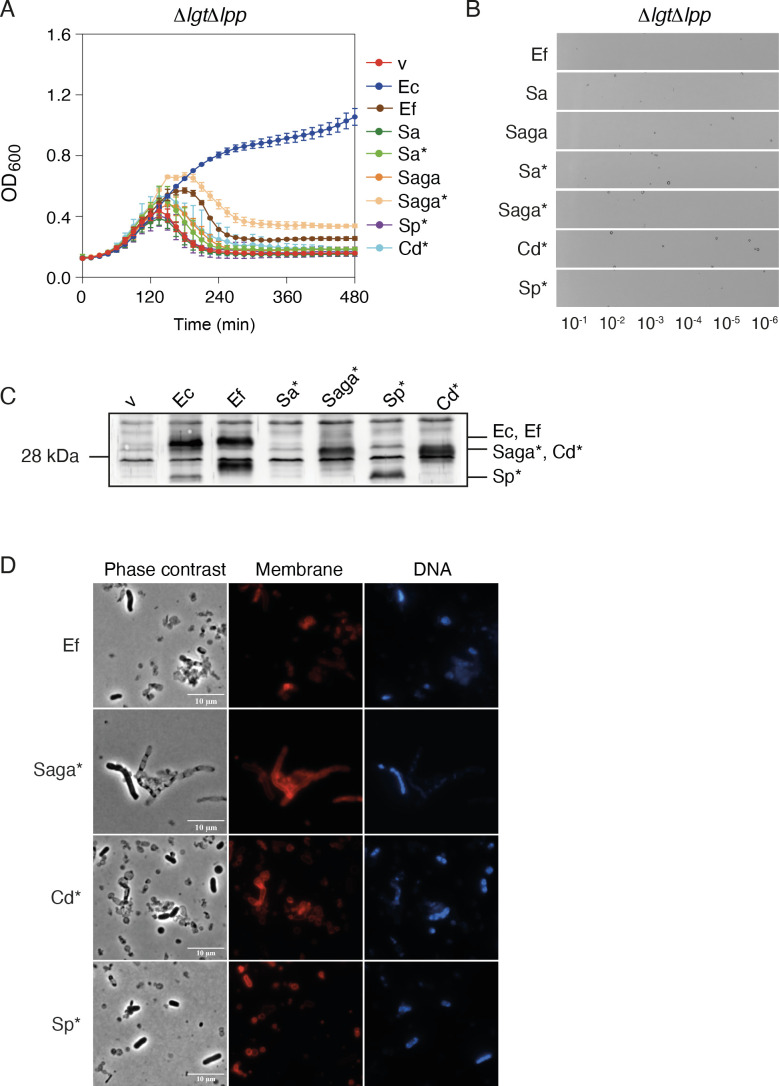
Lgt homologs from monoderm bacteria are not functional in *E. coli.* (**A**) Growth of Δ*lgt*Δ*lpp* (SLEC68) containing plasmids with *lgt* genes from Firmicutes in the presence of 0.2% D-glucose and 5 mM IPTG. Asterisk represents codon-optimized *lgt* genes from *S. aureus* (Sa), *S. agalactiae* (Saga), *C. difficile* (Cd), and *S. pneumoniae* (Sp) for expression in *E. coli*. Ef, *E. faecalis*. Assays were completed in duplicates; error bars indicate standard deviation from the mean. (**B**) Spot dilution assay of strains grown in the presence of 0.2% D-glucose and 5 mM IPTG corresponding to *T*_0_ of the growth curve shown in panel **A**. (**C**) Detection of Lgt-FLAG_3_ with anti-Flag antibodies upon expression of *lgt-flag_3_* from low-copy plasmid pAM238 in *E. coli* MG1655^Q^ by addition of 5 mM IPTG. Total cell lysates corresponding to 0.1 OD_600_ units were loaded to each lane. (**D**) Microscopy images of fixed cells corresponding to *T*_270_ of the growth curve. Sample with Lgt_Sa* was not included due to lack of protein detection. Membranes were stained with lipophilic dye FM4-64X (red), and nucleoids were stained with Hoechst 2333 (blue). Scale bar: 10 μm.

We next asked whether Lgt from Firmicutes can restore growth and viability of *E. coli*Δ*lgt*Δ*lpp*. Growth was not restored when *lgt* of *Enterococcus faecalis* (Ef), *Staphylococcus aureus* (Sa), *Streptococcus agalactiae* (Saga), *Streptococcus pneumoniae* (Sp), and *Clostridium difficile* (Cd) were expressed in Δ*lgt*Δ*lpp* (Fig. 4A). Codon optimization of *lgt* sequences led to protein detection on Western blot, except for *S. aureus* Lgt (Sa*), and Lgt of *E. faecalis* was produced without codon optimization (Fig. 4C). Cell lysis was observed in all strains producing Lgt (Fig. 4D).

Together, the results show that the capacity of Lgt proteins to function in *E. coli* correlates with the split between Terrabacteria and Gracillicutes, the two main kingdoms of Bacteria (37, 38), and suggests the presence of domains and/or specific amino acids reflecting differences in enzyme function and/or substrate specificity.

### The periplasmic (head) domain is important for Lgt function

The main differences in sequence and predicted structure among Lgt proteins are the arm and head domains (Fig. 1A and 2B). We constructed domain swap proteins between *E. coli* Lgt and the corresponding domains of Lgt from *H. pylori*, *M. tuberculosis*, *S. aureus*, and *E. faecalis* to determine their role in Lgt function.

**Fig 5:**
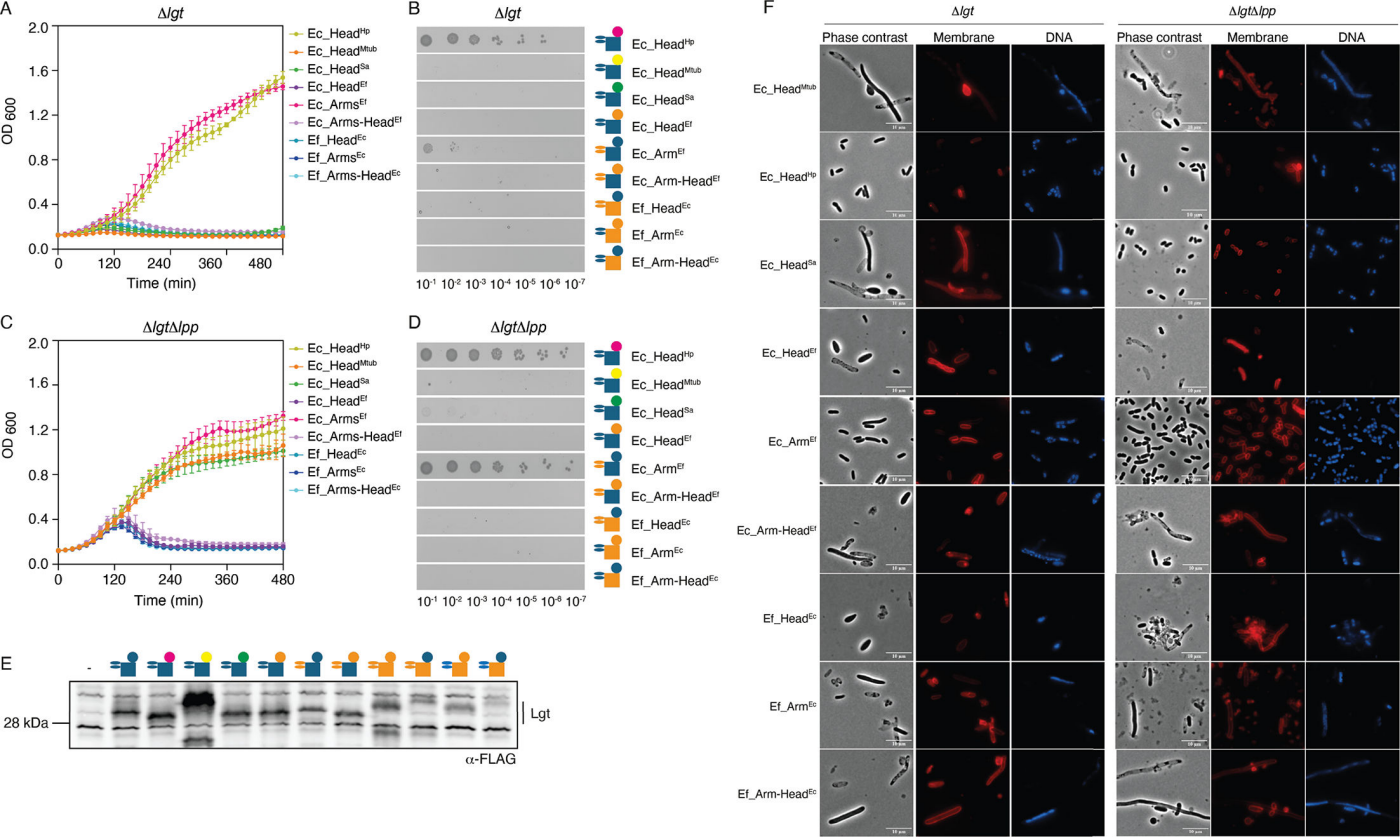
The periplasmic arm and head domains are important for Lgt function. (**A**) Growth of Δ*lgt* (SLEC67) containing *E. coli* Lgt with substituted head domains from *M. tuberculosis* (Head^Mtub^), *H. pylori* (Head^Hp^), *S. aureus* (Head^Sa^), and *E. faecalis* (Head^Ef^), and swap variants between Lgt of *E. coli* and *E. faecalis* encoded on low-copy plasmid pAM238 in 0.2% D-glucose and 5 mM IPTG. Assays were completed in duplicates; error bars indicate standard deviation from the mean. (**B**) Spot dilution assay of Δ*lgt* (SLEC67) derivatives grown on plates with 0.2% D-glucose and 5 mM IPTG corresponding to *T*_0_ of growth curves shown in panel **A**. (**C**) Growth of Δ*lgt*Δ*lpp* (SLEC68) containing the same plasmids as shown in panel **A**. (**D**) Spot dilution assay of Δ*lgt*Δ*lpp* (SLEC68) derivatives grown on plates with 0.2% D-glucose and 5 mM IPTG corresponding to *T*_0_ shown in panel **C**. (**E**) Detection of Lgt-FLAG_3_ on Western blot with anti-Flag antibodies from total cell lysates of MG1655^Q^. Equal cell mass (0.1 OD_600_ units) was loaded per lane. (**F**) Microscopy images of fixed cells corresponding to *T*_270_ of the growth curve. Membranes were stained with lipophilic dye FM4-64X (red), and nucleoids were stained with Hoechst 2333 (blue). Scale bar 10 μm.

Lgt from *H. pylori* (functional in *E. coli*), *E. faecalis* (non-functional, but Lgt produced), and *S. aureus* (Lgt not produced in *E. coli*) have small head domains. The head domain of Lgt of *M. tuberculosis* is similar in size and was selected based on its sequence divergence in the phylogenetic tree (Fig. 1B). All four head swap proteins are produced in *E. coli* (Fig. 5E) including Ec_Head^Sa^, which suggests that the head domain does not determine protein stability. Production of Ec-Head^Hp^ in the two Lgt depletion strains resulted in restoration of growth in liquid medium and colony formation even in the presence of Lpp (Fig. 5A and C). As expected, the morphology of Δ*lgt* and Δ*lgt*Δ*lpp* producing Ec_Head^Hp^ is similar to the strains complemented with Lgt of *E. coli* (Fig. 5F). Growth of Δ*lgt*Δ*lpp* was observed with Ec_Head^Mt^ and Ec_Head^Sa^, but not with Ec_Head^Ef^, while small colonies were only observed with Ec_Head^Sa^ (Fig. 5D). Restoration of growth of Δ*lgt*Δ*lpp* corresponds to a morphology similar to wild-type *E. coli* in the case of Ec_Head^Sa^ (Fig. 5F), but filamentous cells and cell lysis were observed with Ec_Head^Mt^ and Ec_Head^Ef^. These results demonstrate that the head domain plays an important role in Lgt function.

### The arm-head-body combination determines species specificity

The arm domain is located at the front cleft constituting the entry site for substrates (23, 25). In order to test its implication in Lgt function, swap proteins were constructed between Lgt from *E. coli* and *E. faecalis*. The latter was chosen based on non-functionality of full-length protein (Fig. 4A and B) and similar protein levels compared to *E. coli* Lgt (Fig. 4C). Arm and head domains were exchanged between Lgt of both species. Lgt from *E. coli* possessing arm-1 and arm-2 of *E. faecalis* Lgt (Ec_Arms^Ef^) restored growth in Δ*lgt* and Δ*lgt*Δ*lpp* (Fig. 5A and C). Few small colonies were formed in Δ*lgt* (Fig. 5B), and normal colonies were observed in Δ*lgt*Δ*lpp* (Fig. 5D). Cell morphology was similar to *E. coli* Lgt (Fig. 5F). Thus, the sequence of the arms is more malleable than the sequence and/or structure of the head domain. *E. coli* Lgt in which both arm and head domains are derived from *E. faecalis* (Ec_Arm-Head^Ef^) was non-functional (Fig. 5A through D), which highlights the importance and specificity of the head domain.

Lgt from *E. faecalis* with arms (Ef_Arm^Ec^), head (Ef_Head^Ec^), and arms-head (Ef_Arm-Head^Ec^) of *E. coli* Lgt did not restore growth nor viability of Δ*lgt* and Δ*lgt*Δ*lpp*, while Lgt proteins were produced; however, the amount of Ef_Arm-Head^Ec^ was lower compared to the other swap proteins, suggesting that this protein is less stable. Cell morphology is comparable between the three constructs; elongated, enlarged cells and cell lysis were observed (Fig. 5F). Contrary to the head domain, the sequence of the arm domain is not strictly correlated with Lgt function, but these domains are not stand-alone functional domains. The results suggests that the central body harboring the enzymatic cavity works together with matching arm and head domains that are species specific.

### Essential residues and their role in Lgt function

**Fig 6:**
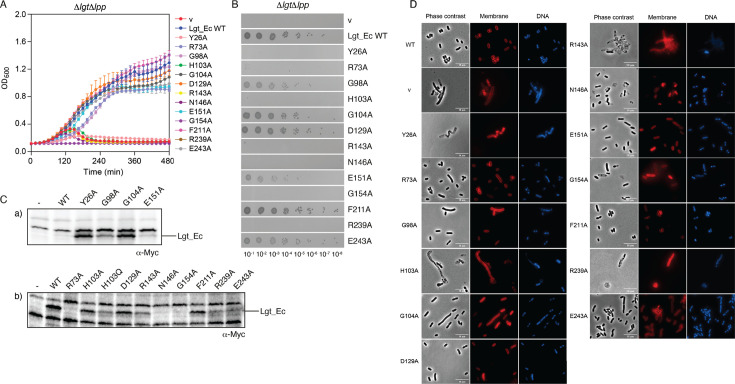
Functional characterization of essential residues in Lgt of *E. coli.* (**A**) Growth of Δ*lgt*Δ*lpp* (SLEC68) with pAM238 plasmids expressing alanine substitution mutants of Lgt of *E. coli* (32) in 0.2% D-glucose and 5 mM IPTG. (**B**) Spot dilution assay grown on plates with 0.2% D-glucose and 5 mM IPTG corresponding to *T*_0_. Assays were performed in duplicate. (**C**) Western blot detection of Lgt-MYC_2_ proteins anti c-Myc antibodies in total cell lysates of MG1655^Q^. Samples were loaded at 0.1 OD_600_U (**A**) and in at 0.25 OD_600_U (**B**). (**D**) Microscopy images of fixed cells corresponding to *T*_270_ of the growth curve. Membranes were stained with lipophilic dye FM4-64X (red), and nucleoids were stained with Hoechst 2333 (blue). Scale bar: 10 μm.

Sixteen residues are conserved between Lgt of *E. coli* and the Lgt proteins from pathogenic species (Fig. 1A). Of these, 12 were shown to be essential, H103 is essential but poorly conserved, and D129 is not essential (23, 24, 32). To obtain a complete and detailed insight into the role of conserved residues in Lgt function, we took advantage of strain Δ*lgt*Δ*lpp*, our previously reported Lgt variants, and newly constructed R73A and F211A mutants. All site-directed alanine mutant proteins were produced in *E. coli* (Fig. 6C), but quantities varied consistently with previous reports (32). Variants Y26A, G98A, G104A, and E151A were produced in higher quantity compared to wild-type Lgt and other homologs (Fig. 6C). A growth delay was observed in the presence of Lgt-E151A in wild-type *E. coli*, which suggests a dominant negative effect over endogenous Lgt (Fig. S4). A slight effect on growth was also observed for G98A, but less for G104A and Y26A (Fig. S4). Lower level of Lgt protein was observed for R73A, N146A, G154A, R239A, and E243A (Fig. 6C), suggesting that these variants are less stable.

Lgt with Y26A (TM1), H103A (TM1), R143A (TM4), N146A (TM4), G154A (loop between TM4 and head domain), and R239A (TM6) did not restore growth (Fig. 6A) and did not form colonies in Δ*lgt*Δ*lpp* (Fig. 6B). These non-functional Lgt variants cause cell lysis (Fig. 6D), confirming the essentiality of these six residues (23, 32). Interestingly, growth of Δ*lgt*Δ*lpp* with Lgt-H103Q differs from Lgt-H103A and reached mid-exponential phase without a rapid decrease in optical density (Fig. S5). This Lgt variant is either more stable than H103A, or glutamine can compensate for histidine in the enzymatic reaction.

Lgt with R73A, G98A, G104A, D129A, E151A, F211A, and E243A grow similar as wild-type Lgt in Δ*lgt*Δ*lpp* (Fig. 6A). Lgt-R73A, with R73 located on top of TM2 close to arm-2, and Lgt-G98A, with G98 located between arm-2 and TM3, are slightly delayed in growth (Fig. 6A). Colonies were formed with all Lgt variants that complement growth, except with R73A (Fig. 6B), suggesting a role for R73 that is different from N146, G154, R239, and E243. Lgt-E151A formed smaller colonies compared to wild-type *E. coli* (Fig. 6B), probably correlated with a dominant negative effect on growth as observed in Fig. S4.

**Fig 7:**
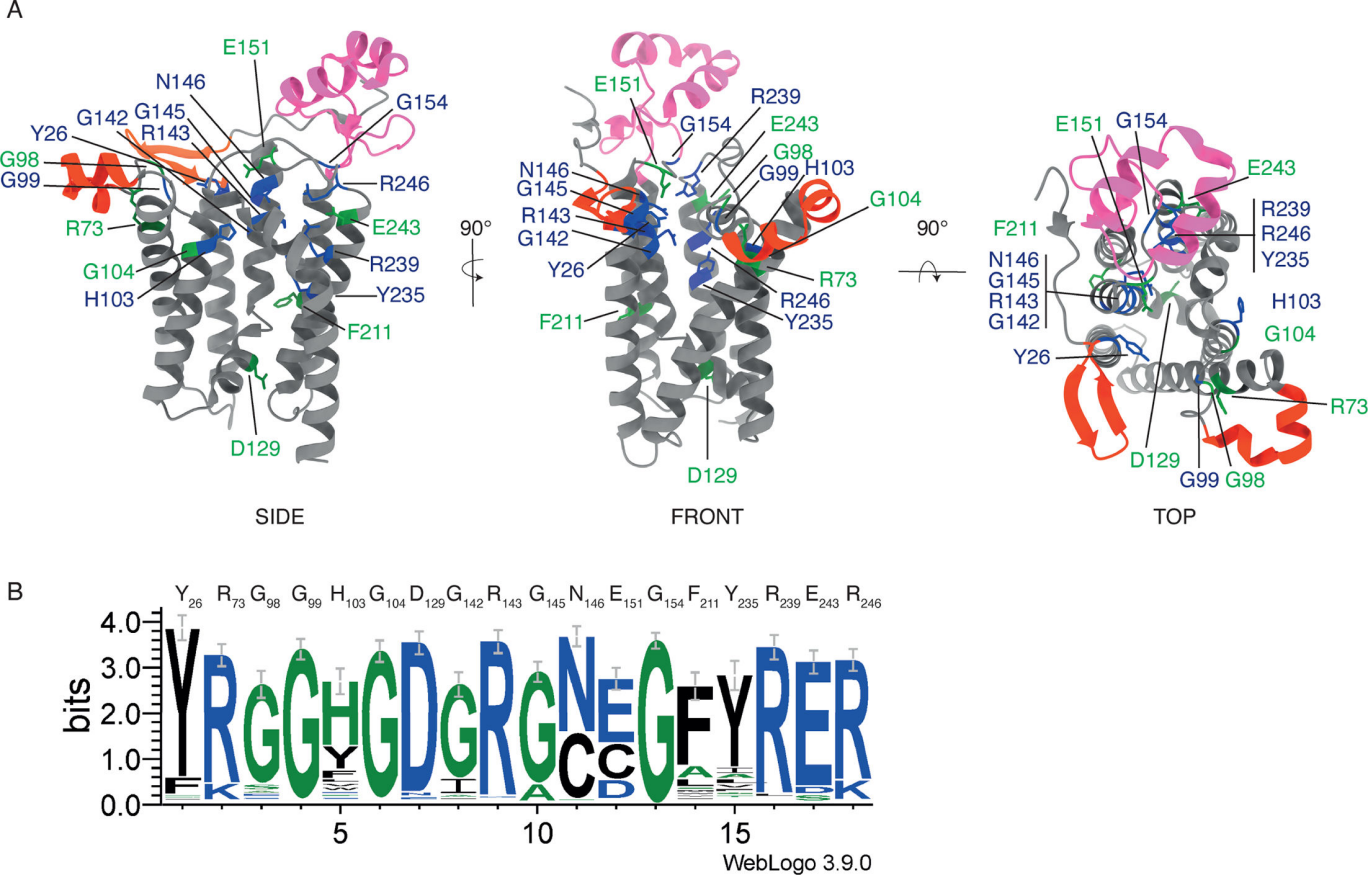
Structural conservation of essential and proposed catalytic residues in Lgt of proteobacteria. (**A**) Eighteen conserved residues—16 conserved residues shown in Fig. 1A and G98 and H103—are depicted on the X-ray crystal structure PDB 5azc of *E. coli* Lgt. Residues Y26, H103, R143, N146, G154, and R239 are essential (blue), and R73, G98, G104, D129, E151, F211, and E243 are not essential (green). Residues G99, G142, G145, Y235, and R246 were shown essential for viability by Mao et al. (23). (**B**) WebLogo of 18 conserved residues shown in panel **A**, based on alignment of 179 Lgt proteins from 160 selected genomes presented in Fig. S8. Residues refer to *E. coli* Lgt.

Five glycine residues are identified among the conserved amino acids (Fig. 1A). Their position close to potential catalytic residues H103 (G104), R143 (G142), and N146 (G145), and the connection between TM4 (body) and head domain (G154) (Fig. 7A) suggest a role in conformational changes upon substrate binding and catalysis (25, 39).

Next, to obtain clues about amino acids implicated in Lgt function other than catalysis that may determine substrate specificity, we identified residues in Lgt that are unique for Proteobacteria but absent in Firmicutes (Fig. S6). We performed separate MSA of the 14 Lgt proteins from Proteobacteria and 7 Lgt proteins from Firmicutes (Fig. 1A) and identified the conserved residues in each group. Out of 22 residues that are unique in Proteobacteria, five were shown to be essential (23); Y30, Y80, M100, and S101 are located at the front cleft close to arm-2, and G263 is located in the bend of TM7 (Fig. S6). These results suggest that entry of substrates through the front cleft is guided by arm-2 via interaction with specific residues that determine substrate specificity, and that bending of TM7 by G263 provides flexibility for catalysis or controlled exit of DAG-modified products together with the gating residue R239 (25).

### Lgt of *Bacteroides thetaiotaomicron* is not functional in *E. coli*

**Fig 8:**
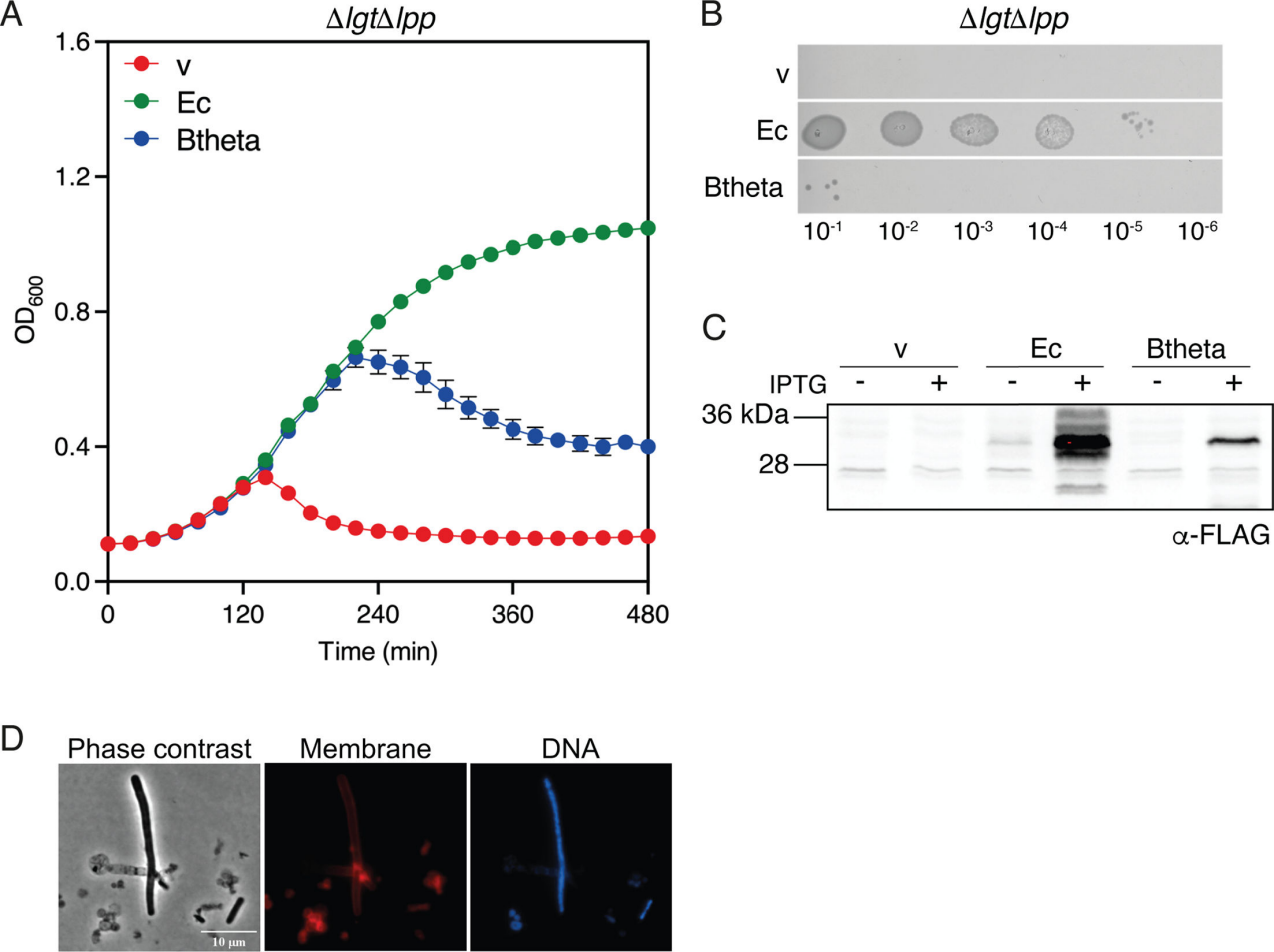
Lgt from *B. thetaiotaomicron* is not functional in *E. coli.* (**A**) Growth of Δ*lgt*Δ*lpp* (SLEC68) expressing *lgt* from *B. thetaiotaomicron* from P*_lac_* on pAM238 in 0.2% D-glucose and 5 mM IPTG. (**B**) Spot dilution assay on plates with 0.2% D-glucose and 5 mM IPTG corresponding to cultures of *T*_0_. (**C**) Western blot detection of Lgt-FLAG_3_ with anti-FLAG antibodies in MG1655^Q^ lysates grown in the absence (−) and presence (+) of 5 mM IPTG. (**D**) Microscopy images of fixed cells corresponding to *T*_270_ of the growth curve. Membranes were stained with lipophilic dye FM4-64X (red), and nucleoids were stained with Hoechst 2333 (blue). Scale bar 10 μm.

One of the major challenges in antimicrobial development is to protect the microbiota when treating bacterial infections caused by pathogenic species. *Bacteroides thetaiotaomicron* (*B. theta*) is an obligate anaerobic diderm bacterium and a prominent member of the human gut microbiota (40). Sequence alignment of Lgt from *B. theta* with Lgt of *E. coli* shows low conservation, although an AF2 structural model resembles Lgt of *E. coli* (Fig. S7). Three changes in *B. theta* Lgt are observed among the 16 conserved residues of *E. coli*: G142I, Y235I, and R246K. Lgt of *B. theta* did not restore growth of Δ*lgt*Δ*lpp* in liquid medium and did not form colonies on glucose/IPTG plates. *B. theta* Lgt is produced at lower levels compared to *E. coli* Lgt (Fig. 8) but in similar quantities as shown for other non-functional Lgt homologs or swap constructs. Together, these data indicate that *B. theta* Lgt is not functional in *E. coli* (Fig. 8).

### Lgt is evolutionary conserved in all bacteria and absent from archaea

We next applied a phylogenomic approach to explore the conservation of Lgt at a larger evolutionary scale. We first searched the refseq_genomes database for prolipoprotein phosphatidylglycerol diacylglyceryl transferase–encoding genes using the Pfam domain PF01790. At the time of analysis (February 2021), 14,664 hits were identified in 12,956 out of 13,512 different genomes (Table S2). Lgt is found in bacteria, including those that lack a cell wall, and is absent from archaea, although the presence of lipoproteins has been reported (41, 42). Recent work identified two genes not homologous to their bacterial counterparts in the archaeon *Haloferax volcanii* that have distinct non-redundant roles in lipoprotein modification. Simultaneous deletion of both genes abolished diphytanylglyceryl modification of Cys_+1_ in lipoproteins (43).

Approximately 1,300 genomes have two *lgt* copies, in agreement with current literature, as shown, for example, in *Streptomyces coelicolor* (44) and in *M. smegmatis* (45), and in members of Firmicutes (e.g., the *B. cereus* group), where one *lgt* gene is frequently located on a plasmid (46). Plasmids with a copy of *lgt* also encode lipoproteins implicated in DNA conjugation via a type IV secretion machinery (Table S2). Some species have up to four *lgt* genes (*Deinococcus, Marinilactibacillus*), while bacteria with small genomes lack a *lgt* gene, for example, endomutualists (Buchnera) or endopathogens (Orientia) (Table S2).

To obtain information on conserved domains and residues, we analyzed 179 Lgt sequences from 160 genomes covering all phyla (35), of which 45% were annotated in the refseq_genome database. Lgt from *E. coli* K12, *H. pylori* 26695, and *M. tuberculosis* H37Rv were included as reference sequences. Phylogenetic data show that essential residues in Lgt are highly conserved (Fig. S8).

Based on complementation results reported here and in the literature, we identified Y26, H103, R143, N146, G154, and R239 among the 18 conserved residues as being absolutely required for the function of *E. coli* Lgt (Fig. 8A and B) (23, 32). Residues G99, G142, G145, Y235, and R246 are essential for viability (23), where G99 and R246 are highly conserved and less variable (Fig. 8B; Fig. S8). Of the functional variants, G104 and D129 are more conserved than G98, E151, and F211. R73 and R246 are replaced by lysine in several Lgt proteins (Fig. S8). In some species, H103 is replaced by Y, W, or F, even in closely related bacteria; H103 is thus not absolutely conserved. In more distantly related Lgt proteins, N146 and E151 are replaced by C or C/D, respectively (Fig. 8B; Fig. S8). In *M. smegmatis*, one Lgt protein (MSMEG_3222) is the major diacylglyceryl transferase and contains N146, while the second homolog (MSMEG_5408) possesses Y26H and N146C with presumed low Lgt activity (45). Finally, one subgroup contains amino acids that greatly vary from the conserved Lgt motif and even lack the fully conserved R143 and R239 residues (Fig. S8). These include *Deinococci*, *Acidimicrobiales*, *Anaerolineales*, *Acidobacteriales*, and *Myxococcales*. The absence of these residues, which are proposed to bind the glycerol headgroup of the phospholipid and perform a gating function (R239) (25), suggests that these Lgt enzymes use a different acyl substrate or perform a diacylglyceryl transferase reaction through a different mechanism.

## DISCUSSION

The lipoprotein modification pathway has gained potential as a target for antibacterial development due to the essentiality of the enzymes in Proteobacteria and their orientation in the membrane providing easy access to small inhibitory molecules (16, 17). Lgt catalyzes the first committed step in lipoprotein modification and is, as we report here, evolutionarily and structurally conserved among bacteria and absent from archaea. Our complementation studies show that Lgt is functionally conserved among diderm bacteria but differs from Lgt of monoderm bacteria. Lgt from *E. faecalis*, *S. agalactiae*, *S. pneumoniae*, and *C. difficile* are not functional in *E. coli*, and Lgt from *S. aureus* was not detected in *E. coli* lysates even when *lgt* was codon-optimized for expression. Early work, however, showed that plasmid-encoded Lgt of *S. aureus* complements a *lgt*(ts) mutant of *E. coli* (31). The G104S mutation in *lgt*(ts) leads to less efficient Lgt activity, which is reflected by the delay in growth that may be compensated by low amounts of Lgt from *S. aureus* at the restrictive temperature.

Subtle differences are observed in growth and viability with Lgt from Proteobacteria. Lgt of *S. enterica* serovar Thyphimurium is closely related to *E. coli* Lgt but only fully complement the *E. coli* Lgt depletion strain in the absence of Lpp. This observation is somewhat puzzling. *S. enterica* possesses two copies of *lpp* that are both required for virulence (47). Both Lpp-1 and Lpp-2 are highly conserved, as well as to Lpp of *E. coli*, suggesting substrate compatibility between the two bacterial species. Currently, there is no evidence that *S. enterica* possesses multiple copies of *lgt*. Of the Lgt proteins tested from Proteobacteria, Lgt of *H. influenzae*, *P. aeruginosa*, *H. pylori*, *N. gonorrhoeae*, and *A. baumannii*are able to compensate loss of viability of the *E. coli* Lgt depletion strain in the absence of Lpp. *L. interrogans* Lgt restores cell morphology but only poorly viability in the absence of Lpp. Furthermore, few essential residues are conserved in *L. interrogans* Lgt, including R143 and R239, but several conserved residues are not. Most striking is the substitution of N146 by cysteine. A second cysteine residue (position W153 in Lgt of *E. coli*) is close enough to form a disulfide bond, perhaps stabilizing *L. interrogans* Lgt. This is in line with the low-level protein detection for N146A due to instability, resulting in non-functionality of this Lgt variant.

We showed that the periplasmic head domain is important for Lgt function in cells where high abundant Lpp is present. El Rayes et al. demonstrated that a disordered linker between the lipid-modified cysteine and the mature folded part of the protein is important for correct sorting to the outer membrane via the Lol machinery in *E. coli* (48). We hypothesize that the flexible linker domain in preprolipoprotein interacts with the head domain, with a role for G154 connecting TM4 and the head domain, to position itself in the catalytic cavity by its signal peptide. *E. coli* Lgt, where the arm domain is exchanged by the corresponding sequence from *E. faecalis* Lgt, is functional, in particular in the absence of Lpp, suggesting that it may fulfill a general function in accommodating both substrates: phosphatidylglycerol and lipobox containing preprolipoproteins.

The arm and head domains are the most diverse, with unique residues located close to arm-2 at the front cleft. For this region, unlike the head domain, a high probability of protein-protein interaction is predicted, which confirms docking studies between Lgt and lipopeptide substrate (25). Shared and essential residues are likely involved in enzyme catalysis, either as catalytic residues or involved in substrate binding. Literature studies, including *in silico* docking experiments with a short lipopeptide substrate, propose an important role for histidine 103 in catalysis (24, 25). Our data show that histidine 103 is poorly conserved and is often substituted by amino acids that cannot serve as a catalytic base in the Lgt reaction. Furthermore, substitution of histidine 103 by glutamine or asparagine, residues acting as weak nucleophiles, results in loss of Lgt function (Fig. S5) (24, 32). Its implication may involve protein substrate binding and correct positioning of preprolipoprotein in the catalytic cavity, while other strictly conserved residues with a more severe phenotype when mutated are directly involved in catalysis. R143 and R239 are involved in positioning the phosphate group of PG in the catalytic site; however, while R143 may have a direct role in catalysis, R239 may assure stability of the enzyme-PG interaction. We propose that glycine residues provide flexibility to neighboring essential and catalytic residues that correctly position PG and preprolipoprotein in the central cavity. While the X-ray structure and molecular modeling provide insight into the interaction of Lgt and PG (23, 25), a high-resolution structure of Lgt with its preprolipoprotein substrate, including a membrane-spanning signal peptide, is needed to understand the precise reaction mechanism.

Two inhibitors of *E. coli* Lgt have been reported. Cyclic peptides were synthesized and selected for Lgt binding and tested in an *in vitro* Lgt assay based on cleavage of glycerol-3-phosphate by G3P dehydrogenase after its initial release from a G1P-G3P mixed PG (21). Cyclic peptide G2824 containing natural and non-natural amino acids was identified as Lgt inhibitor, also acting on *P. aeruginosa* and *A. baumannii* when outer membranes were chemically permeabilized. No effect was observed on *S. aureus* cells, suggesting species specificity. A second report described a small molecule targeting Lgt using an elegant envelope stress reporter assay in which envelope damage leads to release of fluorescent GFP (22). Resistant mutants against MAC-0452936 mapped to Lgt with amino acid substitutions in V109, A37, or G138. V109 is conserved in Proteobacteria, except for ε-Proteobacteria, and is located next to unique residue G108 on TM3, close to essential residues histidine 103, glycine 104, and leucine 106 (23). MAC-0452936 inhibits *E. coli* Lgt activity *in vitro*, and overproduction of Lgt reduces its activity. As for peptide G2428, no inhibitory effect was observed with MAC-0452936 on *S. aureus*.

The functional diversity between Lgt of bacteria belonging to different phyla is likely due to differences in substrate specificity both for the preprolipoprotein and the phospholipid. In general, the lipid moiety reflects the acyl chain composition of membrane phospholipids, although phospholipids in *H. pylori* contain mainly C14 fatty acids, while model lipoproteins are modified by C16:C18 (49). In Proteobacteria, the main phospholipids are PG, cardiolipin (CL), and phosphatidylethanolamine (PE) with C14 to C20 saturated- and unsaturated fatty acids (50). The strains of Firmicutes tested here do not possess PE and often have lysyl-PG and galactosyl- (staphylococci) or glucosyl-diacylglycerol (enterococci) as membrane components (51). Branched-chain fatty acids are also present in Bacilli, including *S. aureus* (52), which influence thickness and fluidity of the membrane, consequently affecting integral membrane protein function. Membranes of *E. faecalis* do not contain branched fatty acids but possess non-saturated carbon chains, like *E. coli* (53). This fact might explain the difference we observed in heterologous protein production in *E. coli*, where Lgt of *E. faecalis* is produced but not Lgt from *S. aureus*.

Future biochemical characterization of Lgt will elucidate how diacylglyceryl transfer takes place and will highlight the basis of substrate specificity between bacteria. Furthermore, the restricted functional conservation between pathogens suggests the potential for developing narrow spectrum antibiotics targeting Lgt.

## MATERIALS AND METHODS

### Bacterial strains and growth conditions

LB or LB agar (LBA) was used for bacterial growth, with spectinomycin (50 mg/L), 0.2% or 4% L-arabinose, 0.2% D-glucose, and 5 mM IPTG added when required. Strains are listed in Table S1. Viability of Δ*lgt* and Δ*lgt*Δ*lpp* derivatives was assessed by colony-forming units (CFU) after growing strains overnight, washing, and diluting in LB and growth for 2 h in LB/spec without sugar at 37°C to deplete Lgt. Cultures were then serially diluted and spotted onto LBA plates. Growth for complementation and microscopy analyses was performed in 96-well plates, with OD_600_ measurements over 18 h. Results were analyzed using GraphPad Prism v10.

### Plasmid constructions

pCHAP9246 (32) was used to generate complementation plasmids, replacing the c-myc_2_-tag with a flag_3_-tag (54). Plasmids were transformed into *E. coli* DH5α and selected for spectinomycin resistance. Further plasmid modifications involved inserting *lgt* genes from different species using EcoRI/XbaI digestion and Gibson assembly. Primers are listed in Table S3. For head domain swap constructions, plasmids were linearized by PCR using outward primers from the flanking regions of the head domains. The head-domain encoding regions were then inserted. The swap constructs, composed of *lgt* sequences from *E. coli* and *E. faecalis*, as well as *lgt* sequences of *C. difficile*, *C. trachomatis*, *S. pneumoniae*, and *L. interrogans*, were ordered as DNA fragment (GeneStrands, Eurofins) (Table S4) and cloned between EcoRI/XbaI sites in plasmid SLP14 (34). All plasmids were confirmed by sequencing (Table S2).

### SDS-PAGE and western blot

Cell pellets were resuspended in Leammli sample buffer (31.5 mM Tris [pH 6.8], 1% SDS, 10% glycerol, 0.005% bromophenol blue), heated, and total cell lysates were loaded onto an 12% SDS-PAGE gel. Proteins were transferred to nitrocellulose membranes, blocked either in 5% milk, 0.05% Tween 20 in TBS (α-MYC; Sigma C3956) or in 5% BSA, 0.5% Tween 20 in PBS (α-FLAG; Sigma F3165), and incubated for 1 h at room temperature with primary antibody. Blots were washed in TBS with 0.05% Tween 20 or in PBS with 0.5% Tween 20 and incubated with secondary HRP coupled antibodies (donkey anti-rabbit (NA934V) in TBS-T or sheep anti-mouse (NA931V) in PBS-T (GE Healthcare). Detection was performed by chemiluminescence using ECL Femto (Thermo Scientific) and imaged on a ChemiDoc imager (Bio-Rad).

### Phase contrast and fluorescence microscopy

Cells were harvested after 270 min of growth and fixed for 15 min at room temperature in a mixture of formaldehyde (2.8%) and glutaraldehyde (0.04%), washed in PBS, and stained with Hoechst 2333 (10 μg/mL) and FM4-64X (0.2 μg/mL) for DNA and membrane visualization, respectively. Cells were immobilized on agar pad (1% agarose in H_2_O), imaged using a Zeiss Axio Observer microscope, and further analyzed with MicrobeJ v5.13I.

### Sequence identification of Lgt proteins

Lgt sequences from WHO Priority, ESKAPE, and CDC Threat pathogens were obtained from the SyntTax database (30) and compared with multiple sequences using the SyntTax web tool to select a representative strain. Lgt homologs were searched in RefSeq using Pfam PF01790 domain with HMM profile and score threshold suggested by Pfam, and sequences were verified using tBlastN and SyntTax. The plasmid database WASPS (46) was explored for the presence of extrachromosomal plasmid copies of *lgt*.

### Multiple sequence alignments and phylogenomic analysis

Alignments were made with Geneious Prime (2024.0.7) and MAFFT v7.481 (55) using the L-INS-I option and were trimmed with BMGE-1.12 (56). A maximum-likelihood tree was generated with IQ-TREE v2.0.7 (57), and the evolutionary model LG+F+G4 was chosen by ModelFinder (58) according to Bayesian Information Criterion (BIC).

### AlphaFold2 structural prediction

The X-ray crystal structure of *E. coli* Lgt (PDB 5azc) and Lgt homologous sequences were used for AlphaFold2 predictions (33). Essential residues were mapped using ChimeraX (v1.10), and model confidence and error were evaluated with pIDDT and PAE graphs.

## SUPPLEMENTAL MATERIAL

Supplemental material mbio.00600-26-s0001.docx Supplemental figures and tables.

Table S2 mbio.00600-26-s0002.xlsx Lgt proteins identified in ref_seq genomes.

ASM does not own the copyrights to Supplemental Material that may be linked to, or accessed through, an article. The authors have granted ASM a non-exclusive, world-wide license to publish the Supplemental Material files. Please contact the corresponding author directly for reuse.
