# Supplementary material for: Arm and head domain in highly conserved lipoprotein modification enzyme Lgt determine functional diversity among bacterial pathogens
Source: mBio. 2026 May 13;17(6):e00600-26. doi: 10.1128/mbio.00600-26 (PMC13251419; doi:10.1128/mbio.00600-26)
Supplement: Supplemental material — Supplemental figures and tables. [file mbio.00600-26-s0001.docx]

**Supplementary data**

Legood *et al*. entitled: Arm and head domain in highly conserved lipoprotein modification enzyme Lgt determine functional diversity among bacterial pathogens.

**S1 Fig.1. Alignment of Lgt from *C. trachomatis* (A) and *L. interrogans* (B) with Lgt of *E. coli*.** Conserved motifs H_103_HGL and Lgt signature are highlighted in blue and green, respectively, similar to Fig. 1. Corresponding structural domains (arms and head) and transmembrane segments 1 to 7 are shown.

**S2 Fig. 2. Confidence metrics and predicted error for Lgt model structures.** AF2 pIDDT and PAE graphs for 16 Lgt proteins shown in Fig. 2. Low confidence and predicted errors in the Lgt sequences are indicated by asterisk and arrows, respectively.

**S3 Fig. 3. Complementation of Δ*lgt*Δ*lpp* by basal level expression of *E. coli* *lgt*.** (A) Growth of SLEC68 in LB with 0.2% D-glucose in the presence of absence of 5 mM IPTG. (B) Detection of *E. coli* Lgt-FLAG_3_ by anti-FLAG antibodies in MG1655^Q^ background. v; empty vector, Ec; *E. coli* Lgt. (C) Microscopy images of fixed cells corresponding to T_270_ of the growth curve. Membranes were stained with lipophilic dye FM4-64X (red) and nucleoids with Hoechst 2333 (blue). Scale bar 5 μm.

**S4 Fig. 4. Effect on growth of highly produced Lgt variants.** Growth of *E. coli* MG1655^Q^ strain with pAM238 constructs carrying lgt alanine mutants were induced with 5 mM IPTG was recorded in 96-well plates in a TECAN spectrophotometer. Controls were empty plasmid and *E. coli* lgt (left panel) compared to Y26A, G98A, G104A and E151A Lgt (right panel).

**S5 Fig. 5. Effect of substitution of H103 by glutamine on growth of Δ*lgt*Δ*lpp*.** Strains were grown in LB with D-glucose (0.2%) in the presence of absence of 5 mM IPTG.

**S6 Fig. 6. Structural position of unique and conserved residues in Lgt of Proteobacteria.** Essential residues conserved in 14 Lgt proteins from Proteobacteria depicted in Fig. 1. Essential residues are shown on the X-ray crystal structure of *E. coli* Lgt (5azc); essential residues (red), non-essential residues (green), non-determined residues (black) are indicated. Unique residues Y30, Y80, M100, S101 and G263 are conserved in Proteobacteria and absent from Firmicutes and are essential for Lgt function in *E. coli* as reported by Mao *et al.* (1).

**S7 Fig. 7. Sequence alignment and structure prediction of Lgt from *B. thetaiotaomicron*.** (A) Protein sequence alignment of Lgt from *E. coli* and *B. theta* by ClustalO. Among the 16 conserved residues (Fig. 1) 13 are conserved in *B. theta* Lgt (red) and 3 (G142I, Y235I, R246K) are variable. B) AF2 model of Lgt from *B. theta* and (C) corresponding pIDDT and pAE graphs, asterisk and arrows indicate low confidence and high error in predicted structure. Arm (orange) and head (pink) domains are shown and TMS 1-7 indicated in blue.

**S8 Fig. 8. Phylogeny of 179 Lgt proteins from 160 genomes representing all phyla highlight the degree of conservation of essential residues in Lgt.** The maximum-likelihood tree was inferred from an alignment of 179 Lgt sequences. The 18 residue Lgt motif obtained from functional studies: Y_26_-R_73_-G_98_-G_99_-H_103_-G_104_-D_129_-G_142_-R_143_-N_145_-N_146_-E_151_-G_154_-F_211_-Y_235_-R_239_-E_243_-R_246_ was analyzed for the degree of conservation and the identification of amino acid substitutions. The scale bar represents the average number of substitutions per site. *E. coli* K12, *H. pylori* 26695 and *M. tuberculosis* H37Rv as references.

**ST 1. Strains and plasmids used in this study.**

**ST 2. Lgt proteins identified in ref_seq genomes.** Added as a separate file.

**ST 3. Primers used in this study.**

**ST 4. Synthetic *lgt* gene fragments used in this study.**

**ST 5. Amino acids exchanged in Lgt swap proteins.**

**
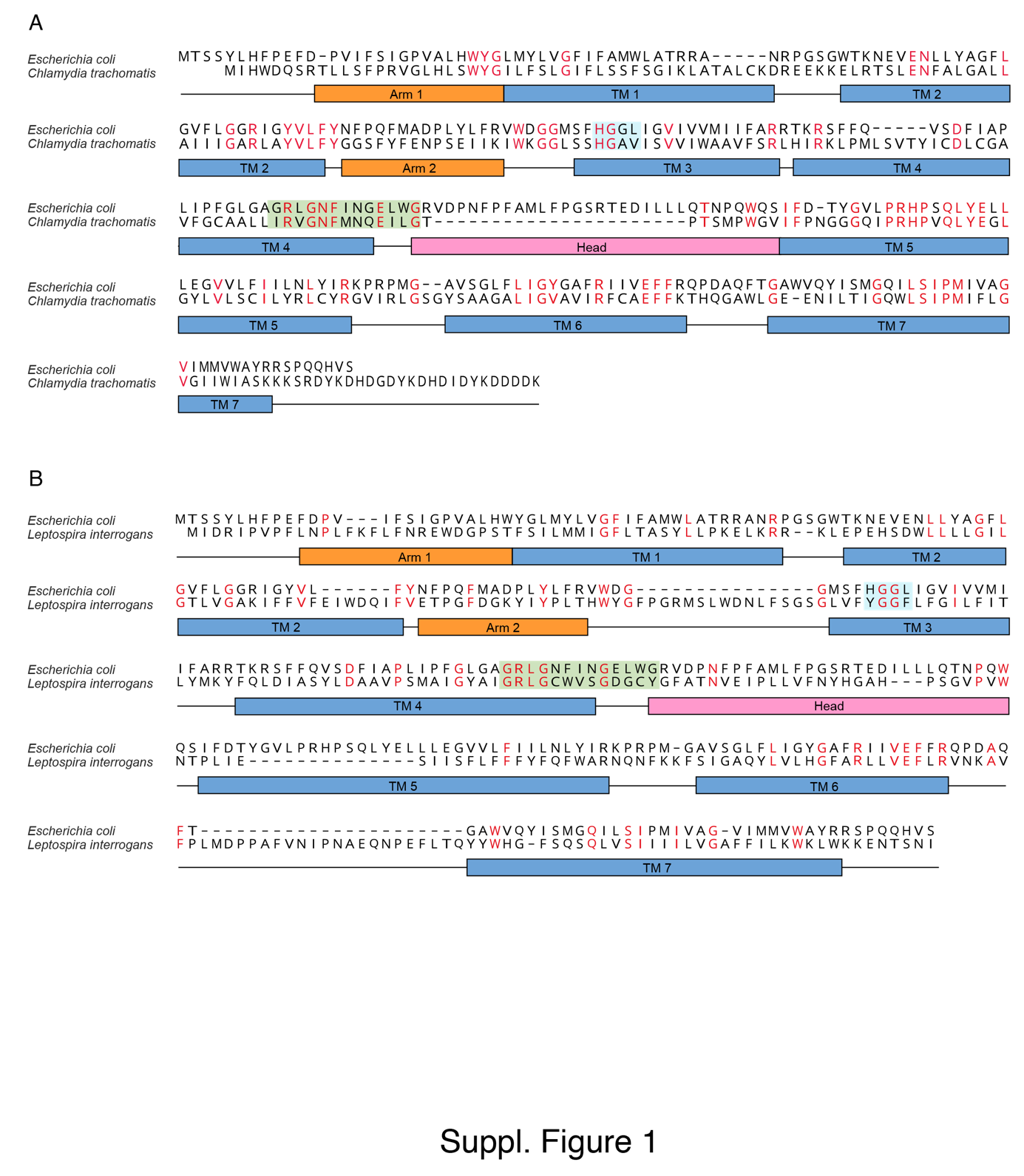
**

**
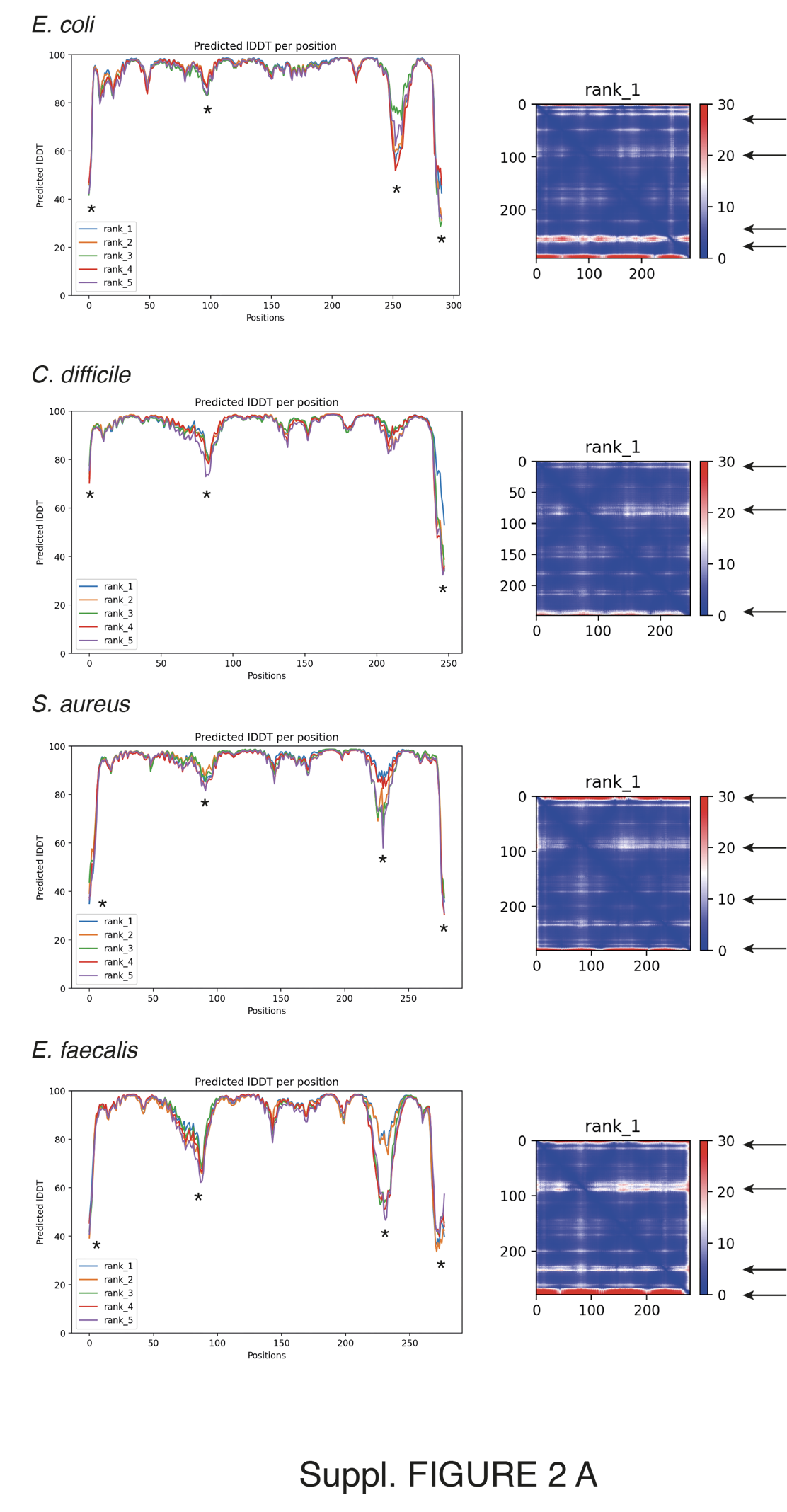
**

**
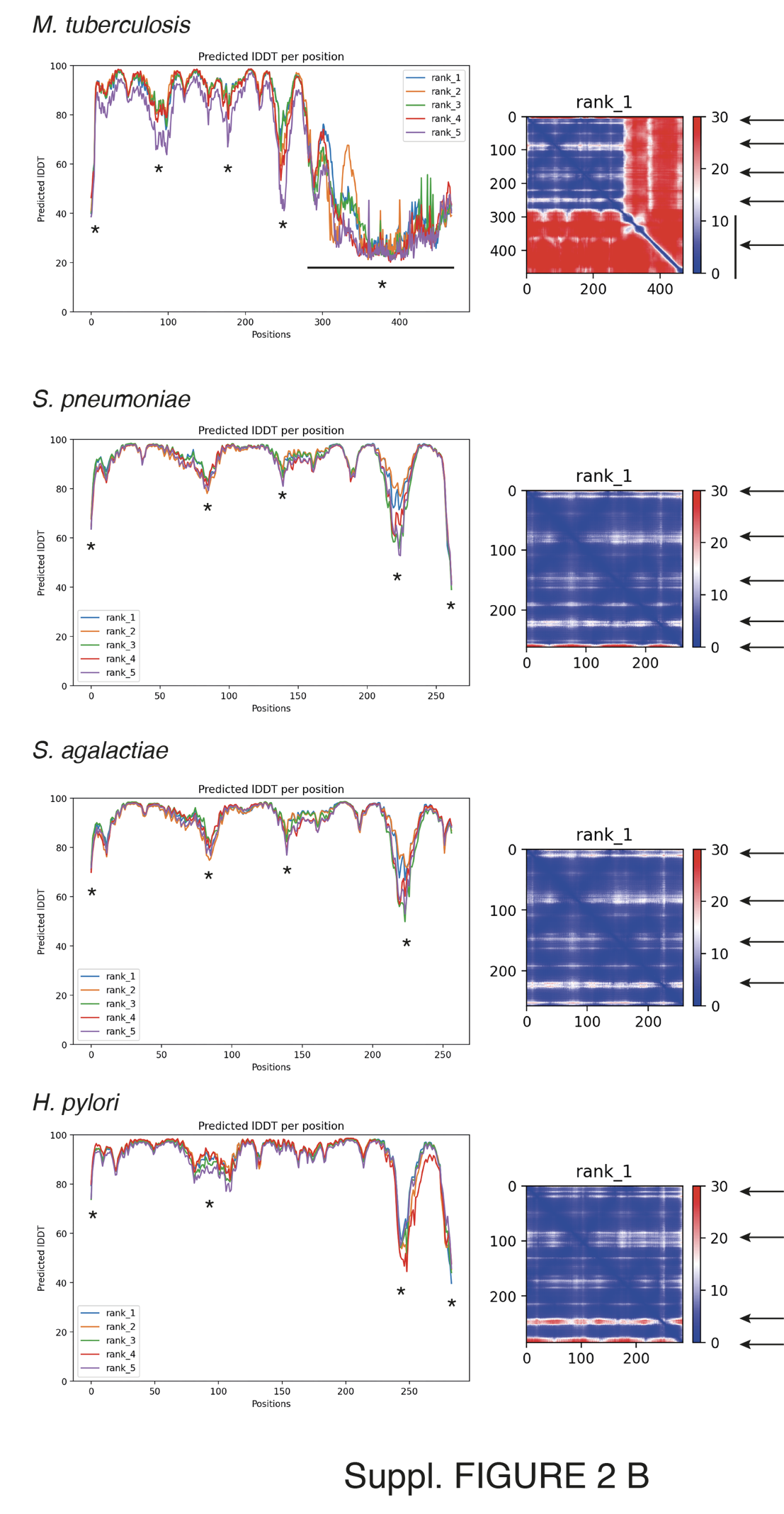
**

**
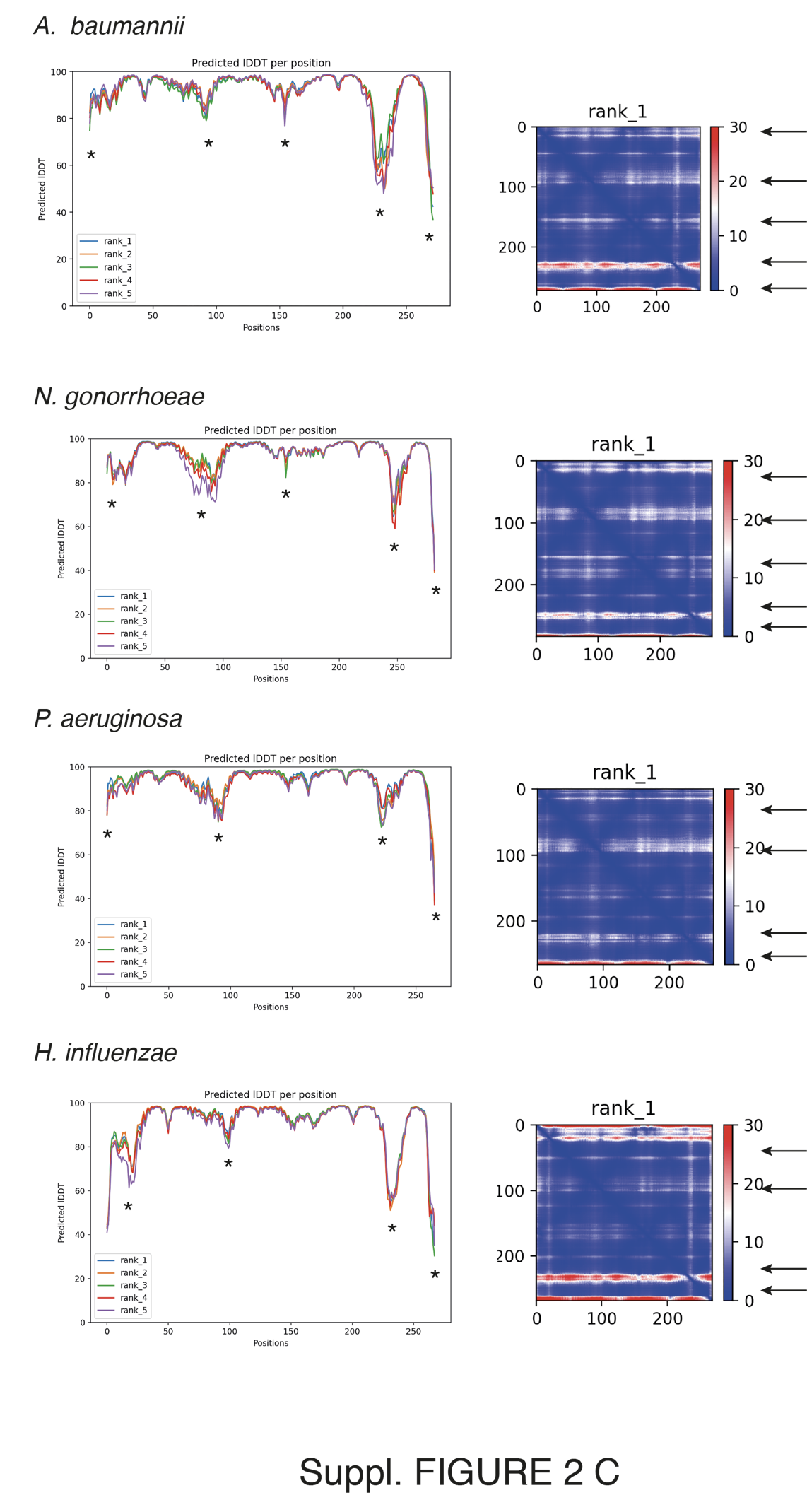
**

**
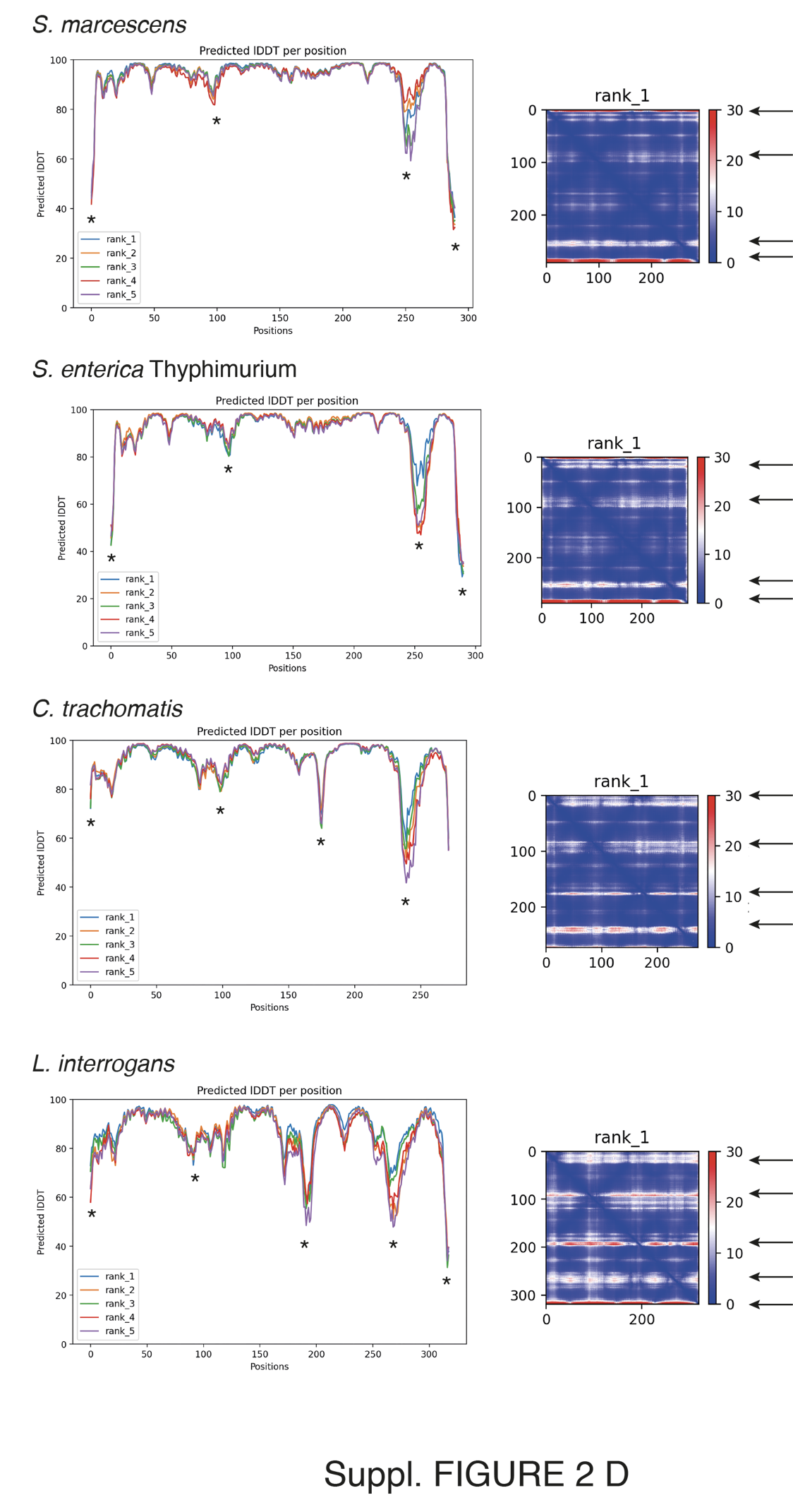
**

**
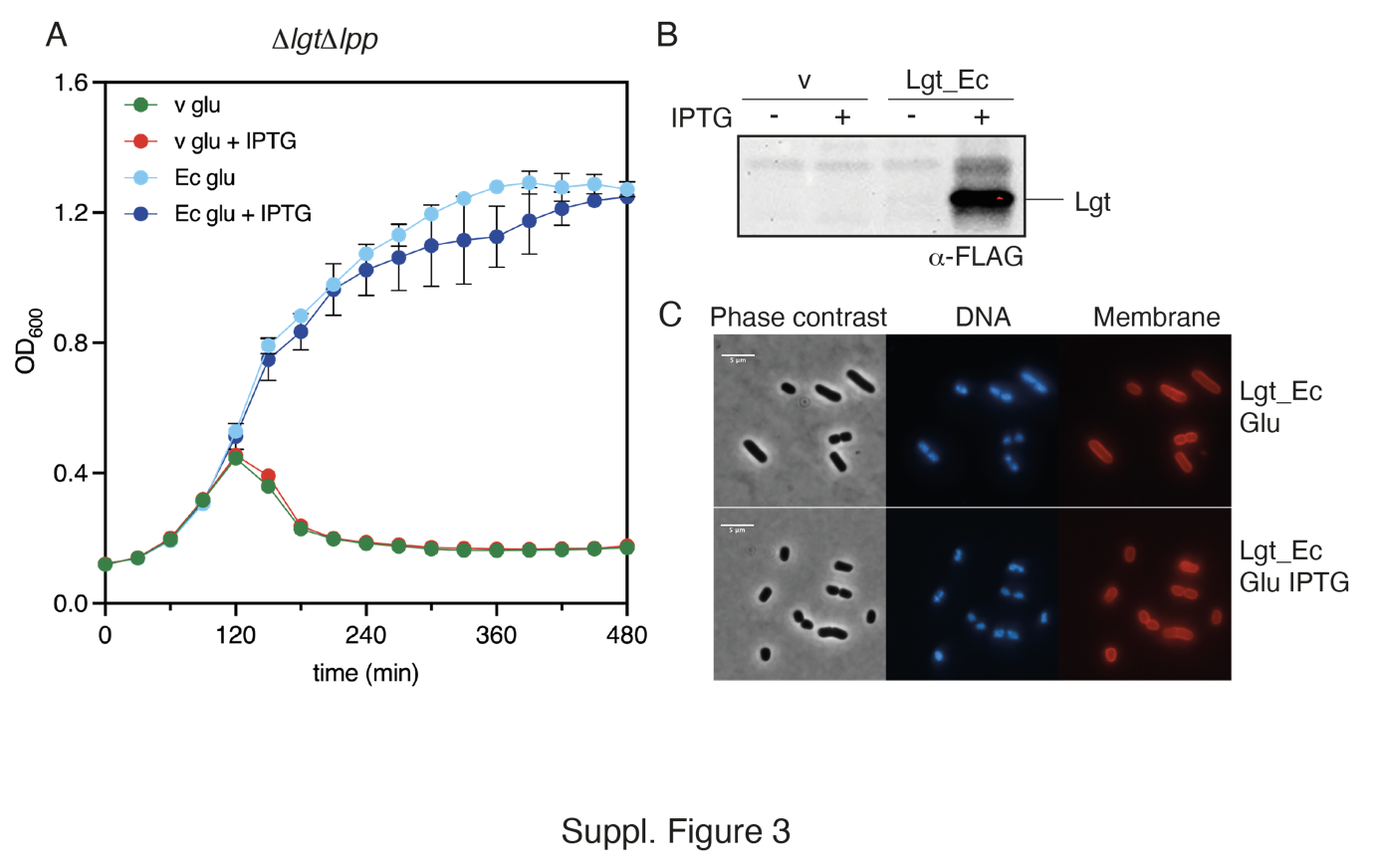
**

**
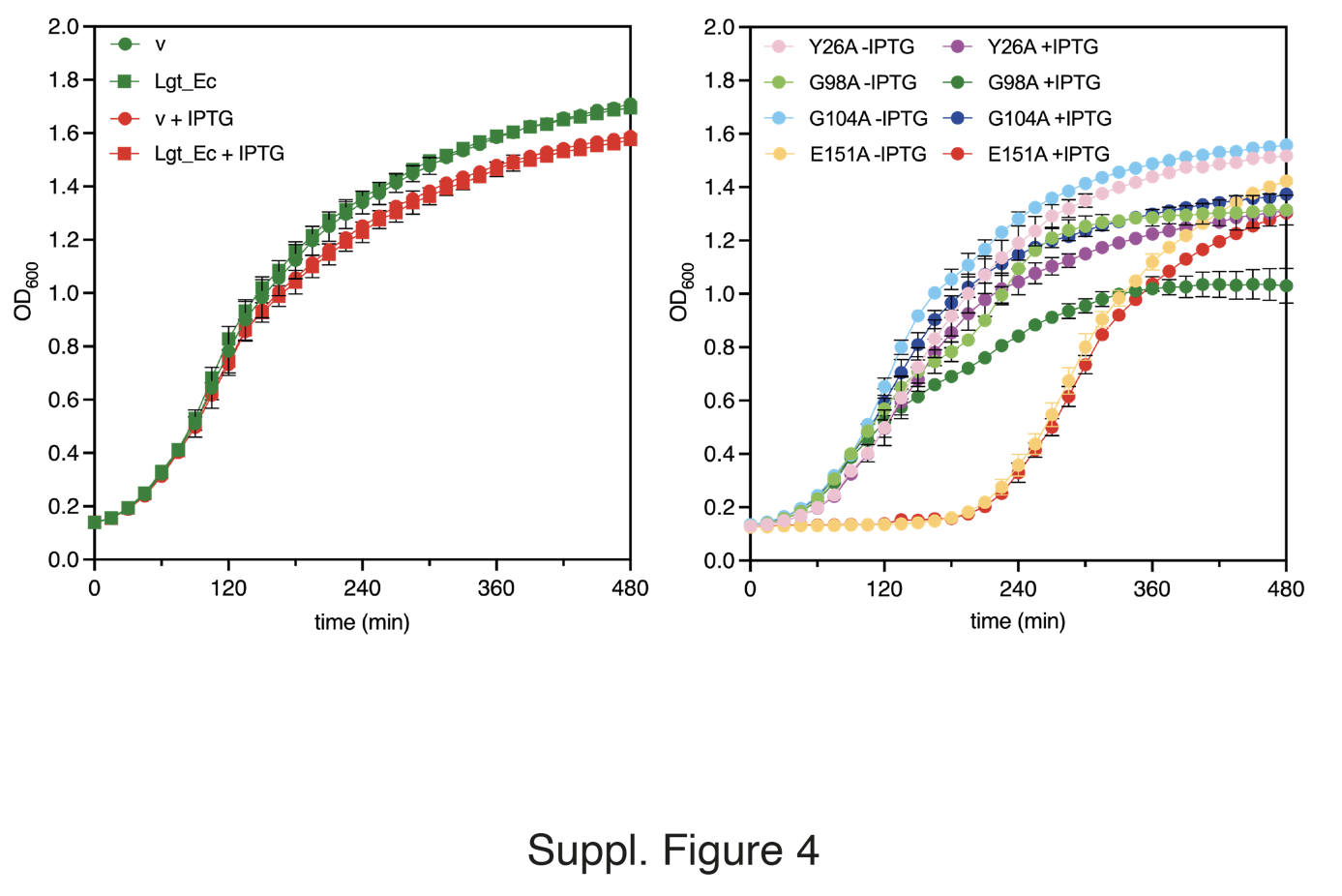
**

**
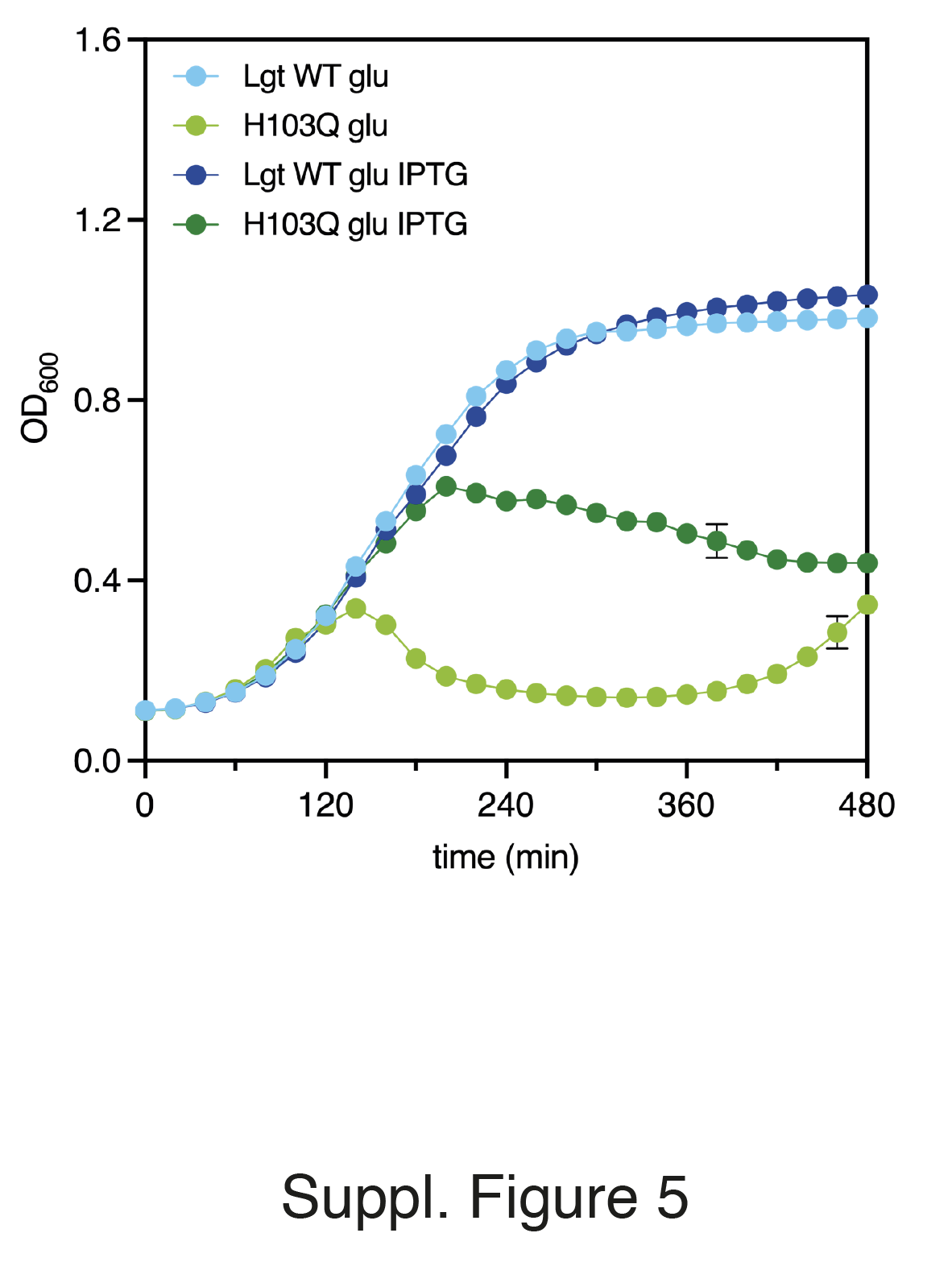
**

**
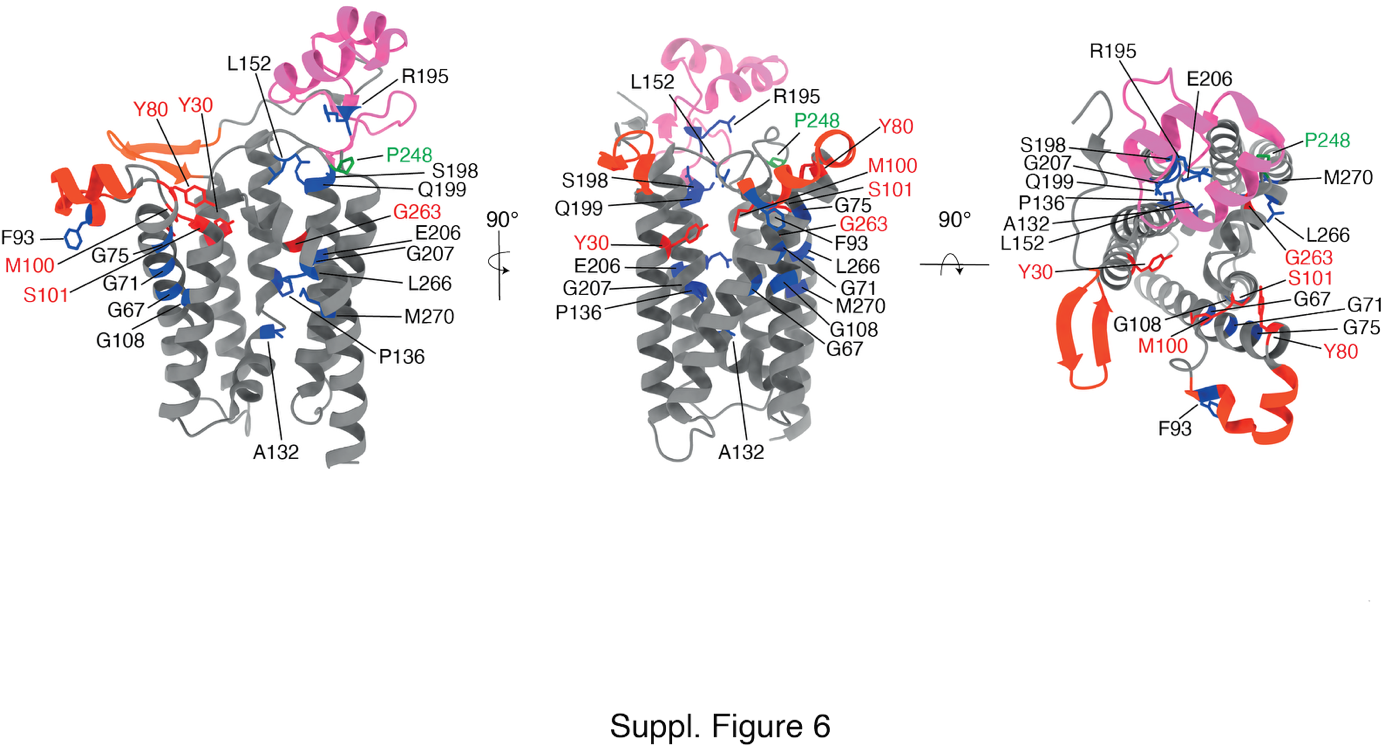
**

**
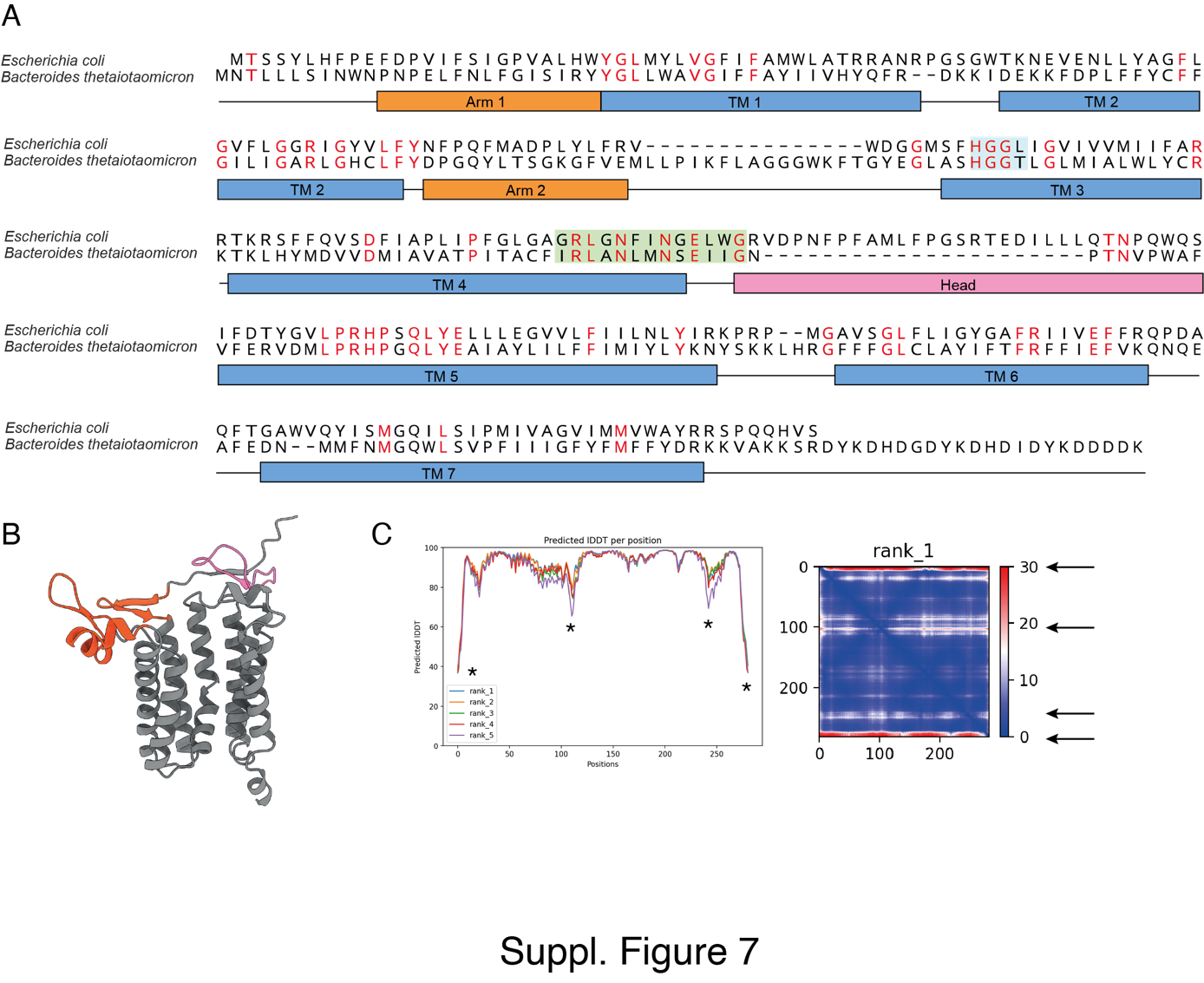
**

**
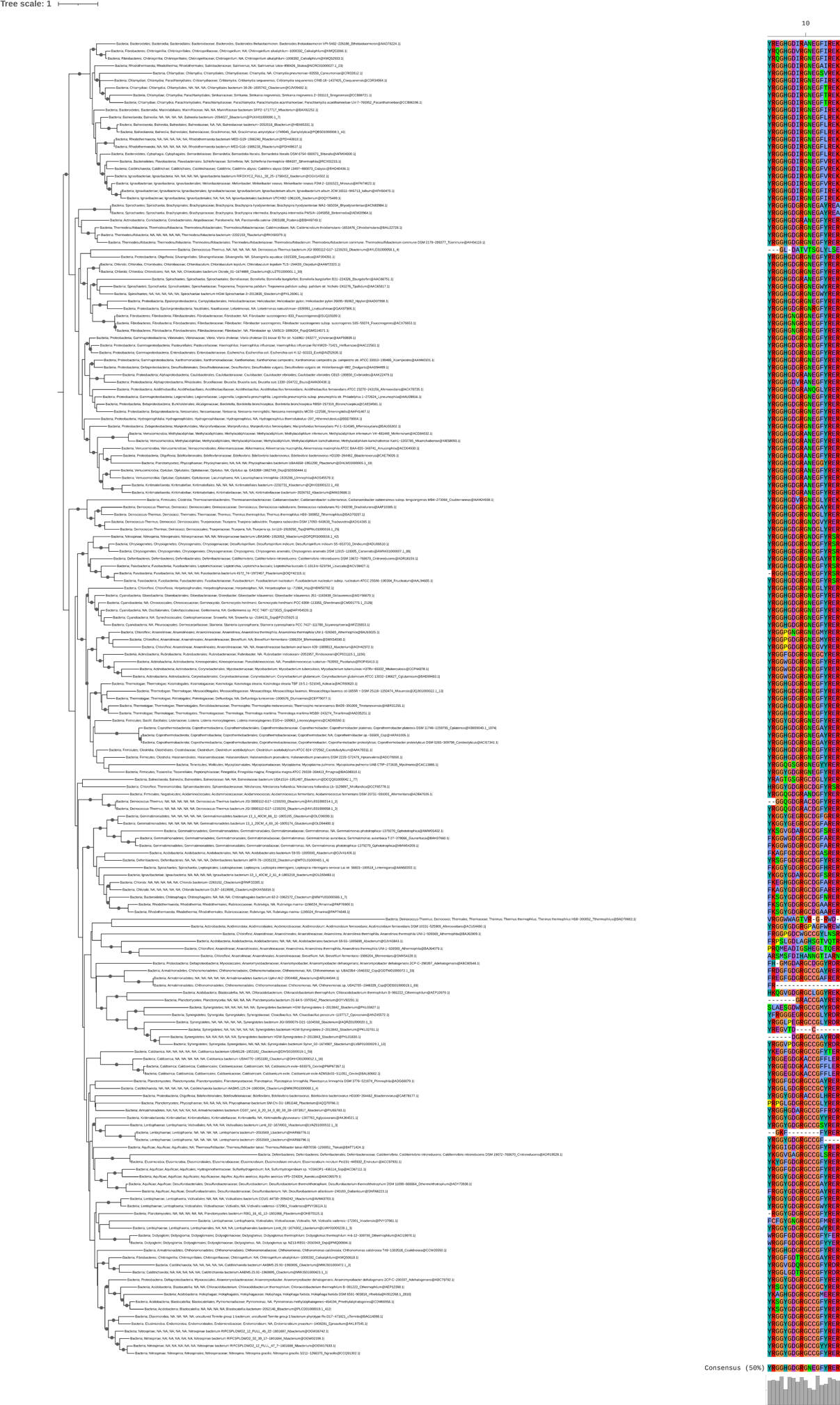
**

Suppl. Figure 8

**Suppl. Table 1. Strains and plasmids used in this study.**

| Strains | Description | Genotype | Reference |
| --- | --- | --- | --- |
| BW25113 |  | *E. coli* K-12 *lacI^q^ rrn*B_T14_ Δ*lacZ*_WJ16_ *hsdR514* Δ*araBAD*_AH33_ Δ*rhaBAD*_LD78_ | (1) |
| MG1655 *lac*I^q^ |  | F- lambda- *ilvG*- *rfb*-50 *rph*-1 | Lab collection |
| SLEC67 | Δ*lgt*^c^ | Lgt depletion in MG1655 *lac*I^q^ | (2) |
| SLEC68 | Δ*lgt*^c^Δ*lpp* | Lgt depletion in MG1655 *lac*I^q^ *lpp*::Tn10 | (3) |
| Plasmids |  |  |  |
| pAM238 |  | pSC101 origin, p_Lac_ promoter, Spc^r^ | (4) |
| SLP14 | pAM238-lgt_Ec-flag3 | *lgt* of *E. coli* with *flag*_3_ tag in pAM238 | (3) |
| SLP17 | pAM238-lgt_Pa-flag3 | *lgt* of *P. aeruginosa* | This study |
| SLP20 | pAM238-lgt_Ab-flag3 | *lgt* of *A. baumannii* | This study |
| SLP34 | pAM238-lgt_Se-flag3 | *lgt* of *S. enterica* serovar Thyphimurium | This study |
| SLP35 | pAM238-lgt_Sa-flag3 | *lgt* of *S. aureus* | This study |
| SLP36 | pAM238-lgt_Saga-flag3 | *lgt* of *S. agalactiae* | This study |
| SLP38 | pAM238-lgt_Hi-flag3 | *lgt* of *H. influenzae* | This study |
| SLP39 | pAM238-lgt_Ef-flag3 | *lgt* of *E. faecalis* | This study |
| SLP40 | pAM238-lgt_Ng-flag3 | *lgt* of *N. gonorrhoeae* | This study |
| SLP41 | pAM238-lgt_Hp-flag3 | *lgt* of *H. pylori* | This study |
| SLP42 | pAM238-lgt_Sa*-flag3 | Codon optimized sequence *lgt* of *S. aureus* | This study |
| SLP43 | pAM238-lgt_Saga*-flag3 | Codon optimized sequence *lgt* of *S. agalactiae* | This study |
| SLP45 | pAM238-lgtHead^Hp^-flag3 | Head domain of *lgt* of *H. pylori* in Lgt of *E. coli* | This study |
| SLP46 | pAM238-lgtHead^Mtub^-flag3 | Head domain of *lgt* of *M. tuberculosis* in Lgt of *E. coli* | This study |
| SLP47 | pAM238-lgtHead^Sa^-flag3 | Head domain of *lgt* of *S. aureus* in Lgt of *E. coli* | This study |
| pAP3 | pAM238-lgt_Btheta-flag3 | *lgt* of *B. thetaiotaomicron* | This study |
| pAP11 | pAM238-lgt_Sm-flag3 | Codon optimized sequence *lgt* of *Serratia marcensens* | This study |
| pAP12 | pAM238-lgt_Ec-myc2 H103A | Alanine substitution of H103 in *lgt* of *E. coli* | This study |
| pAP13 | pAM238-lgt_Ec-myc2 R73A | Alanine substitution of R73 in *lgt* of *E. coli* | This study |
| pAP14 | pAM238-lgt_Cd*-flag3 | Codon optimized sequence *lgt* of *Clostridioides difficile* | This study |
| pAP15 | pAM238-lgt_Sp*-flag3 | Codon optimized sequence *lgt* of *Streptococcus pneumonia* | This study |
| pAP17 | pAM238-lgt_Ec Arm^Ef^-Head^Ef^-flag3 | Arm and Head domains of *E. faecalis* in Lgt of *E. coli* | This study |
| pAP18 | pAM238-lgt_Ef Head^Ec^-flag3 | Head domain of *E. coli* in Lgt of *E. faecalis* | This study |
| pAP19 | pAM238-lgt_Ef Arm^Ec^-flag3 | Arm domains of *E. coli* in Lgt of *E. faecalis* | This study |
| pAP20 | pAM238-lgt_Ef Arm^Ec^-Head^Ec^-flag3 | Arm and Head domains of *E. coli* in Lgt of *E. faecalis* | This study |
| pAP21 | pAM238-lgt_Ec-myc2 F211A | Alanine substitution of F211 in *lgt* of *E. coli* | This study |
| pAP22 | pAM238-lgt_Ec Head^Ef^-flag3 | Head domain of *E. faecalis* in Lgt of *E. coli* | This study |
| pAP23 | pAM238-lgt_Ec Arm^Ef^-flag3 | Arm domains of *E. faecalis* in Lgt of *E. coli* | This study |
| pAP24 | pAM238-lgt_Ct*-flag3 | Codon optimized sequence *lgt* of *Clamydia trachomatis* | This study |
| pAP25 | pAM238-lgt_Li*-flag3 | Codon optimized sequence *lgt* of *Leptospira interrogans* | This study |
| pCHAP9246 | pAM238-lgt_Ec-myc2 | *E. coli lgt* with *c-myc*_2_ tag in pAM238 | (5) |
| pCHAP9256 | pAM238- lgt_Ec-myc2 R239A | Alanine substitution of R239 in *lgt* of *E. coli* | (5) |
| pCHAP9260 | pAM238-lgt_Ec-myc2 R143A | Alanine substitution of R143 in *lgt* of *E. coli* | (5) |
| pCHAP9261 | pAM238-lgt_Ec-myc2 E151A | Alanine substitution of E151 in *lgt* of *E. coli* | (5) |
| pCHAP9262 | pAM238-lgt_Ec-myc2 D129A | Alanine substitution of D129 in *lgt* of *E. coli* | (5) |
| pCHAP9280 | pAM238-lgt_Ec-myc2 H103Q | Glutamine substitution of H103 in *lgt* of *E. coli* | (5) |
| pCHAP9282 | pAM238-lgt_Ec-myc2 Y26A | Alanine substitution of Y26 in *lgt* of *E. coli* | (5) |
| pCHAP9283 | pAM238-lgt_Ec-myc2 G98A | Alanine substitution of G98 in *lgt* of *E. coli* | (5) |
| pCHAP9284 | pAM238-lgt_Ec-myc2 G154A | Alanine substitution of G154 in *lgt* of *E. coli* | (5) |
| pCHAP9285 | pAM238-lgt_Ec-myc2 E243A | Alanine substitution of E243 in *lgt* of *E. coli* | (5) |
| pCHAP9286 | pAM238-lgt_Ec-myc2 G104A | Alanine substitution of G104 in *lgt* of *E. coli* | (5) |
| pCHAP9306 | pAM238-lgt_Ec-myc2 N146A | Alanine substitution of N146 in *lgt* of *E. coli* | (5) |

**Suppl. Table 3. Primers used in this study.**

| Primer | Sequence 5’-3’ | Template |
| --- | --- | --- |
| 42 | ATGGTCTTTGTAGTCTCTAGTTTGATTTTCCTTTATTTTTTTAGAATTTTTTGTAGCATACAATAAAATCC | *H. pylori* 26695 |
| 43 | AGCTATGACCATGATTACGAATTCATGAACGCTTGGAATACGATTTATGATCAAT |  |
| 48 | AGCTATGACCATGATTACGAATTCATGCTGACCTATCCTAATATCGATCCG | *A. baumannii* AYE |
| 49 | ATGGTCTTTGTAGTCTCTAGTACTGTTCTTTTGAGGGCCCCA |  |
| 50 | ATGGTCTTTGTAGTCTCTAGTGGCCGCCTTCGGC | *P. aeruginosa* PAO1 |
| 51 | AGCTATGACCATGATTACGAATTCATGCTGACGTATCCCCAGAT |  |
| 126 | CTCGTAAAGCTGTGATGGATGATAATATTGGCCGTTAA | *S. aureus* RN220 |
| 127 | AACGGTGAATTGTGGGGCGGATCGGTGTCACGCGCTTT |  |
| 128 | GGTCTTTGTAGTCTCTAGAATAATATGAAATATGTTGTTCACGACGA | *S. agalactiae* BM110 |
| 129 | GACCATGATTACGGAATTATGATGATTAATCCAGTAGCA |  |
| 130 | GGTCTTTGTAGTCTCTAGAAGAAGTTGTTATTTTTTTCTTTTGTTTCTCC | *E. faecalis* OG1RF |
| 131 | GACCATGATTACGGAATTATGTTAGCTCAAGTAAATTCAATTGCA |  |
| 132 | GGTCTTTGTAGTCTCTAGAGTGCTGTTTTTTCATGCCGA | *N. gonorrhoeae* 27632 |
| 133 | GACCATGATTACGGAATTATGATTATCCATCACCAATTCGATCC |  |
| 134 | GGTCTTTGTAGTCTCTAGAGGAAACGTGTTGCTGCGG | *S. enterica* serovar Thyphimurium ATCC14028 |
| 135 | GACCATGATTACGGAATTATGACCAGTAGCTATCTGCA |  |
| 136 | GCCCCACAATTCACCGTTAAT | SLP14 |
| 137 | CCATCACAGCTTTACGAGCT |  |
| oAP13 | CAGAACATAACCAATTGCTCCCCCGAGGAAGACGC | pAP13 (R73A) |
| oAP14 | GCGTCTTCCTCGGGGGAGCAATTGGTTATGTTCTG |  |
| oAP15 | CGCCAATCAGGCCGCCTGCGAAAGACATGCCGCCG | pAP12 (H103A) |
| oAP16 | CGGCGGCATGTCTTTCGCAGGCGGCCTGATTGGCG |  |
| oAP17 | CAGGTTGAGGATAATTGCCAGCACCACACCTTCC | pAP21 (F211A) |
| oAP18 | GGAAGGTGTGGTGCTGGCAATTATCCTCAACCTG |  |
| 138 | CTCGTAAAGCTGTGATGGTTGAACCACGAACGCCACC | *lgt* head domain *M. tuberculosis* H37Rv |
| 139 | AACGGTGAATTGTGGGGCCGTGAAACCACCATGCCGT |  |
| 140 | CTCGTAAAGCTGTGATGGATGATAATATTGGCCGTTAA | *lgt* head domain *S. aureus* RN220 |
| 141 | AACGGTGAATTGTGGGGCGGATCGGTGTCACGCGCTTT |  |
| 142 | CTCGTAAAGCTGTGATGGATAACGCAACTCATTATCCACCA | *lgt* head domain *H. pylori* 26695 |
| 143 | CTCGTAAAGCTGTGATGGATAACGCAACTCATTATCCACCA |  |
| 144 | CAGCTATGACCATGATTACGAATTCATGAATTCAAATTATTTACTTCTTCC | *H. influenzae* NT31258 |
| 145 | CGTCATGGTCTTTGTAGTCTCTAGATTTTATGACCGCACTTTTG |  |
| 146 | ACAGCTATGACATGATTACGAATTCATGAATACCCTTTTATTATCAATC | *B. thetaiotaomicron* VPI 5482 |
| 147 | CGTCATGGTCTTTGTAGTCTCTAGATTTCTTTGCTACCTTCTTC |  |
| M13F | TAGTCTCTAGAGGAAAC | pAM238 |
| M13R | CAGGAAACAGCTATGAC |  |
| lower_FLAG | AGCTTCTACTTGTCATCGTCATCCTTGTAATCGATGTCATGATCTTTATAATCACCGTCATGGTCTTTGTAGTCT | (6) |
| upper_FLAG | CTAGAGACTACAAAGACCATGACGGTGATTATAAAGATCATGACATCGATTACAAGGATGACGATGACAAGTAGA | (6) |

**Suppl. Table 4. Synthetic *lgt* sequences used in this study.**

| Name | Strain | Sequence 5’-3’ |
| --- | --- | --- |
| *lgt*_Cdiff | Clostridioides difficile strain 020474 R2021 | CTATGACATGATTAC**GAATTC**ATGGATCGCGTAGCATTTACCCTGTTTGGCATCGACATCATGTGGTATGGCATCCTGATGGCATGTGGTATGATTCTGGGTACTCTGATCGCAATCAAAGAAGCAAAACGCGTGGGTATTAAAGATGATGATGTACTGAATATCGCTATTATCGCAATTCCAGTTGGTCTGATCTGTGCACGCATCTATTATGTTGTATTTAATTGGAGCTACTATGCTCAAAATATGTCTCAAATTTTTAATTTTCGTGGTGGTGGCCTGGCTATCCATGGCGGTCTGATTGGTGGCATCCTGGCTGGTTATATCTACACTAAGATTAAAAATATTAACTTCCTGAAAATGGCTGATACTGTTATCCTGGGCATGCCTCTGGCACAAGCTATTGGCCGTTGGGGCAATTTTATTAATGGCGAAGCACATGGCGGTGCTACCAATCTGCCTTGGGGCATCATGGTTGATGGCGTAAAGGTACATCCAACTTTTCTGTATGAGTCCATTTGGGATTTTGGTATTTTTATCGTTCTGCTCCTGTTTCGTAAAAATAAAAAATATGAAGGTCAAGTTATCGTTACTTATATTACTCTGTATTCCATCGGTCGTTTTTTTATCGAGGGCCTGCGCACCGATTCCCTGATGCTGGGTCCACTGCGTATGGCACAAGTTATTTCTCTGATTGGTGTTATTGGTGGCATCATTGCACATGTTTATCTGTCTAAGAAAAATAAACATAATATTTCCGAAGAA**TCTAGA**GACTACAAAGACC |
| *lgt*_Clamydia | NC_000117.1:c284827-284009 Chlamydia trachomatis D/UW-3/CX | CTATGACATGATTAC**GAATTC**GTGATCCATTGGGACCAGTCTCGTACTCTCCTGTCTTTCCCGCGTGTTGGCCTGCATCTGTCCTGGTACGGCATCCTGTTTTCTCTGGGTATTTTTCTGTCCTCTTTTTCTGGCATCAAGCTGGCAACTGCACTGTGTAAAGACCGCGAGGAGAAAAAAGAACTCCGCACGTCTCTGGAAAATTTTGCTCTGGGCGCTCTCCTGGCTATCATCATTGGTGCTCGTCTGGCTTACGTTCTGTTCTATGGCGGTTCCTTTTATTTTGAAAATCCTTCTGAGATTATCAAAATTTGGAAAGGCGGTCTGTCTAGCCATGGCGCTGTGATCTCTGTGGTGATTTGGGCAGCTGTATTTTCTCGTCTGCATATTCGTAAGCTCCCTATGCTGTCTGTAACCTACATTTGCGATCTGTGTGGCGCAGTGTTTGGTTGTGCAGCTCTCCTGATTCGCGTGGGTAACTTTATGAATCAGGAAATTCTCGGTACCCCTACCTCCATGCCTTGGGGTGTGATTTTTCCGAATGGTGGTGGCCAAATCCCTCGCCATCCTGTTCAGCTGTACGAAGGCCTGGGTTATCTGGTGCTGTCTTGTATTCTGTACCGTCTGTGCTATCGTGGTGTTATTCGTCTGGGTTCTGGCTATAGCGCAGCAGGCGCTCTGATTGGCGTAGCAGTAATTCGTTTTTGCGCTGAATTTTTCAAAACCCACCAAGGTGCATGGCTGGGCGAAGAAAACATTCTCACCATTGGTCAATGGCTGTCTATTCCGATGATTTTTCTGGGCGTTGGCATCATTTGGATTGCTAGCAAAAAGAAA**TCTAGA**GACTACAAAGACC |
| *lgt*_Lepto | Leptospira interrogans serovar Copenhageni Fiocruz L1-130 | CTATGACATGATTAC**GAATTC**ATGATTGACCGTATCCCAGTACCGTTTCTCAACCCTCTGTTCAAATTCCTGTTCAATCGTGAATGGGACGGCCCGTCCACGTTTAGCATCCTGATGATGATCGGTTTTCTGACCGCATCTTATCTCCTGCCTAAAGAGCTGAAACGTCGTAAGCTGGAACCAGAACATTCTGATTGGCTCCTGCTCCTGGGCATCCTGGGCACCCTGGTAGGTGCTAAAATCTTTTTCGTCTTTGAAATTTGGGATCAGATCTTTGTAGAGACCCCGGGCTTTGATGGCAAGTATATTTATCCACTCACTCATTGGTATGGTTTTCCTGGCCGTATGTCTCTGTGGGACAATCTGTTTTCTGGTTCCGGCCTCGTATTTTACGGTGGCTTTCTGTTCGGCATTCTCTTTATCACTCTGTATATGAAATACTTTCAACTGGACATTGCATCCTACCTGGACGCTGCGGTTCCATCTATGGCGATCGGTTACGCAATTGGTCGTCTGGGTTGTTGGGTTTCTGGTGACGGTTGTTACGGTTTTGCAACCAATGTTGAAATTCCACTCCTGGTTTTTAATTATCATGGTGCTCATCCGTCCGGTGTACCTGTTTGGAATACTCCACTCATCGAAAGCATCATTTCCTTTCTGTTCTTTTTTTATTTTCAGTTTTGGGCGCGCAATCAAAACTTTAAAAAGTTTTCCATTGGCGCCCAATATCTGGTCCTCCACGGTTTTGCACGTCTCCTGGTGGAATTTCTGCGTGTTAATAAAGCAGTATTTCCTCTGATGGACCCACCTGCATTCGTAAACATTCCGAACGCAGAACAAAATCCTGAATTTCTGACTCAGTATTACTGGCATGGTTTTTCCCAATCCCAACTGGTTTCTATTATTATCATTCTGGTAGGTGCATTTTTTATCCTGAAATGGAAACTCTGGAAAAAAGAAAATACCTCCAACATT**TCTAGA**GACTACAAAGACC |
| *lgt*_Spneumo | Streptococcus pneumoniae strain 11A | CTATGACATGATTAC**GAATTC**ATGCTGGATCCAATTGCTATTCAACTGGGTCCGCTGGCCATTCGTTGGTATGCCCTGTGTATTGTGACGGGCCTGATTCTGGCGGTTTATCTGACCATGAAAGAAGCACCTCGTAAGAAGATCATTCCAGACGATATTCTGGATTTTATCCTGGTAGCTTTTCCGCTGGCTATTCTGGGTGCTCGTCTCTACTATGTTATTTTCCGTTTTGATTACTATTCCCAGAATCTGGGCGAGATTTTTGCCATTTGGAATGGTGGTCTCGCCATTTACGGTGGTCTGATTACTGGTGCTCTGGTGCTCTATATCTTTGCTGACCGTAAACTCATCAATACTTGGGATTTTCTGGATATTGCGGCGCCTAGCGTTATGATTGCTCAAAGCCTGGGTCGTTGGGGTAATTTCTTTAACCAAGAAGCTTATGGTGCAACCGTGGATAATCTGGATTATCTGCCTGGCTTTATCCGTGACCAGATGTATATTGAGGGTAGCTACCGTCAACCGACTTTCCTGTATGAGTCTCTGTGGAATCTGCTGGGCTTTGCCCTGATTCTGATTTTTCGTCGCAAATGGAAGAGCCTCCGCCGTGGTCATATCACGGCCTTTTACCTCATTTGGTATGGTTTCGGTCGTATGGTCATCGAAGGTATGCGCACCGATAGCCTCATGTTCTTCGGCCTGCGTGTGTCCCAATGGCTGTCTGTTGTCCTCATCGGTCTCGGTATCATGATCGTTATTTATCAAAATCGTAAGAAGGCCCCTTACTATATTACTGAGGAGGAAAAC**TCTAGA**GACTACAAAGACC |
| Construct | Plasmid | Sequence 5’-3’ |
| Body_Ec Arm_Ef Head Ef | pAP17 | CTATGACATGATTAC**GAATTC**GCGAAATAACAAGAAATTGTGGTGACAGATGACCAGTAGCTATCTGCATTTTCCGGAGTTTGATTCAATTGCATTTCGCCTTTTCGGTATTCCCGTCTATTGGTACGGCCTGATGTATCTGGTGGGTTTCATTTTTGCAATGTGGCTGGCAACACGACGGGCGAATCGTCCGGGCAGCGGCTGGACCAAAAATGAAGTTGAAAACTTACTCTATGCGGGCTTCCTCGGCGTCTTCCTCGGGGGACGTATTGGTTATGTTCTGTTCTACCAATGGCAAGATTATGTAGACAATCCGATTGAAATTTTCTTTGTCTGGGACGGCGGCATGTCTTTCCACGGCGGCCTGATTGGCGTTATCGTGGTGATGATTATCTTCGCCCGCCGTACTAAACGTTCCTTCTTCCAGGTCTCTGATTTTATCGCACCACTCATTCCGTTTGGTCTTGGTGCCGGGCGTCTGGGCAACTTTATTAACGGTGAATTGTGGGGCGGGCCAGCGACCACCAGACAATTTTTAGAAAATCTCCATTTACCAACGTTTATCATTGATAACATGAATATCAACGGAACGTATCATCAACCATCACAGCTTTACGAGCTGCTGCTGGAAGGTGTGGTGCTGTTTATTATCCTCAACCTGTATATTCGTAAACCACGCCCAATGGGAGCTGTCTCAGGTTTGTTCCTGATTGGTTACGGCGCGTTTCGCATCATTGTTGAGTTTTTCCGCCAGCCCGACGCGCAGTTTACCGGTGCCTGGGTGCAGTACATCAGCATGGGGCAAATTCTTTCCATCCCGATGATTGTCGCGGGTGTGATCATGATGGTCTGGGCATATCGTCGCAGCCCACAGCAACACGTTTCC**TCTAGA**GACTACAAAGACC |
| Body_Ef-Head_Ec | pAP18 | CTATGACATGATTAC**GAATTC**ATGTTAGCTCAAGTAAATTCAATTGCATTTCGCCTTTTCGGTATTCCCGTCTATTGGTATGCAATTATTATTGTTTCAGGGATTGCTTTAGCTGTTTGGCTAAGTAGTCGTGAAGCCGTTCGTGTTGGTTTAAAAGAAGATGATGTCTTTGACTTTATGCTCTGGGGATTACCTGCAGCAATTGTTGGCGCGCGCTTGTATTACGTCGCTTTTCAATGGCAAGATTATGTAGACAATCCGATTGAAATTTTCTTTACTCGAAATGGTGGCTTAGCGATTTATGGTGGTCTTATTGGTGGGGGACTGGCGCTGTTTTTCTTTACGCGTCATCGTTTCATTTCTACATGGACTTTTTTAGATATTGCCGCACCAAGTGTGATTTTAGCGCAAGCAATTGGTCGCTGGGGCAACTTTATGAATCATGAAGCCTATCGCGTTGACCCGAACTTCCCGTTTGCCATGCTGTTCCCTGGCTCCCGTACAGAAGATATTTTGCTGCTGCAAACCAACCCGCAGTGGCAATCCATTTTCGACACTTACGGTGTGCTGCCGCGCCACCCAACATTTTTATATGAATCTGTCTGGAATGTTTTAGGCTTTATCGTTTTAGTGTTATTACGCAAAAAGCCACACTTTTTAAAAGAAGGCGAAGTCTTTTTAGGTTATATAATATGGTATTCTTTCGGCCGCTTCTTCATCGAAGGGTTACGGATGGACAGTTTGTATGCGTTTAGTAATATTCGTGTCTCACAATTGTTGTCTTTAGTGATGTTCGTGGCAGCAATTGTTATTGTGATTGTTCGTAGAAGGAATCCCAATTTGAAATTTTACAATCGGGAGAAACAAAAGAAAAAAATAACAACTTCT**TCTAGA**GACTACAAAGACC |
| Body_Ef-Arm_Ec | pAP19 | CTATGACATGATTAC**GAATTC**ATGTTAGCTCAAGTAAATCCGGTCATTTTCTCAATAGGACCCGTGGCGCTTCACTGGTATGCAATTATTATTGTTTCAGGGATTGCTTTAGCTGTTTGGCTAAGTAGTCGTGAAGCCGTTCGTGTTGGTTTAAAAGAAGATGATGTCTTTGACTTTATGCTCTGGGGATTACCTGCAGCAATTGTTGGCGCGCGCTTGTATTACGTCGCTTTTAATTTCCCGCAGTTTATGGCCGATCCGCTGTATCTGTTCCGTACTCGAAATGGTGGCTTAGCGATTTATGGTGGTCTTATTGGTGGGGGACTGGCGCTGTTTTTCTTTACGCGTCATCGTTTCATTTCTACATGGACTTTTTTAGATATTGCCGCACCAAGTGTGATTTTAGCGCAAGCAATTGGTCGCTGGGGCAACTTTATGAATCATGAAGCCTATGGGCCAGCGACCACCAGACAATTTTTAGAAAATCTCCATTTACCAACGTTTATCATTGATAACATGAATATCAACGGAACGTATCATCAACCAACATTTTTATATGAATCTGTCTGGAATGTTTTAGGCTTTATCGTTTTAGTGTTATTACGCAAAAAGCCACACTTTTTAAAAGAAGGCGAAGTCTTTTTAGGTTATATAATATGGTATTCTTTCGGCCGCTTCTTCATCGAAGGGTTACGGATGGACAGTTTGTATGCGTTTAGTAATATTCGTGTCTCACAATTGTTGTCTTTAGTGATGTTCGTGGCAGCAATTGTTATTGTGATTGTTCGTAGAAGGAATCCCAATTTGAAATTTTACAATCGGGAGAAACAAAAGAAAAAAATAACAACTTCT**TCTAGA**GACTACAAAGACC |
| Body_Ef-Arm_Ec-Head_Ec | pAP20 | CTATGACATGATTAC**GAATTC**ATGTTAGCTCAAGTAAATCCGGTCATTTTCTCAATAGGACCCGTGGCGCTTCACTGGTATGCAATTATTATTGTTTCAGGGATTGCTTTAGCTGTTTGGCTAAGTAGTCGTGAAGCCGTTCGTGTTGGTTTAAAAGAAGATGATGTCTTTGACTTTATGCTCTGGGGATTACCTGCAGCAATTGTTGGCGCGCGCTTGTATTACGTCGCTTTTAATTTCCCGCAGTTTATGGCCGATCCGCTGTATCTGTTCCGTACTCGAAATGGTGGCTTAGCGATTTATGGTGGTCTTATTGGTGGGGGACTGGCGCTGTTTTTCTTTACGCGTCATCGTTTCATTTCTACATGGACTTTTTTAGATATTGCCGCACCAAGTGTGATTTTAGCGCAAGCAATTGGTCGCTGGGGCAACTTTATGAATCATGAAGCCTATCGCGTTGACCCGAACTTCCCGTTTGCCATGCTGTTCCCTGGCTCCCGTACAGAAGATATTTTGCTGCTGCAAACCAACCCGCAGTGGCAATCCATTTTCGACACTTACGGTGTGCTGCCGCGCCACCCAACATTTTTATATGAATCTGTCTGGAATGTTTTAGGCTTTATCGTTTTAGTGTTATTACGCAAAAAGCCACACTTTTTAAAAGAAGGCGAAGTCTTTTTAGGTTATATAATATGGTATTCTTTCGGCCGCTTCTTCATCGAAGGGTTACGGATGGACAGTTTGTATGCGTTTAGTAATATTCGTGTCTCACAATTGTTGTCTTTAGTGATGTTCGTGGCAGCAATTGTTATTGTGATTGTTCGTAGAAGGAATCCCAATTTGAAATTTTACAATCGGGAGAAACAAAAGAAAAAAATAACAACTTCT**TCTAGA**GACTACAAAGACC |
| Body_Ec-Head_Ef | pAP22 | CTATGACATGATTAC**GAATTC**GCGAAATAACAAGAAATTGTGGTGACAGATGACCAGTAGCTATCTGCATTTTCCGGAGTTTGATCCGGTCATTTTCTCAATAGGACCCGTGGCGCTTCACTGGTACGGCCTGATGTATCTGGTGGGTTTCATTTTTGCAATGTGGCTGGCAACACGACGGGCGAATCGTCCGGGCAGCGGCTGGACCAAAAATGAAGTTGAAAACTTACTCTATGCGGGCTTCCTCGGCGTCTTCCTCGGGGGACGTATTGGTTATGTTCTGTTCTACAATTTCCCGCAGTTTATGGCCGATCCGCTGTATCTGTTCCGTGTCTGGGACGGCGGCATGTCTTTCCACGGCGGCCTGATTGGCGTTATCGTGGTGATGATTATCTTCGCCCGCCGTACTAAACGTTCCTTCTTCCAGGTCTCTGATTTTATCGCACCACTCATTCCGTTTGGTCTTGGTGCCGGGCGTCTGGGCAACTTTATTAACGGTGAATTGTGGGGCGGGCCAGCGACCACCAGACAATTTTTAGAAAATCTCCATTTACCAACGTTTATCATTGATAACATGAATATCAACGGAACGTATCATCAACCATCACAGCTTTACGAGCTGCTGCTGGAAGGTGTGGTGCTGTTTATTATCCTCAACCTGTATATTCGTAAACCACGCCCAATGGGAGCTGTCTCAGGTTTGTTCCTGATTGGTTACGGCGCGTTTCGCATCATTGTTGAGTTTTTCCGCCAGCCCGACGCGCAGTTTACCGGTGCCTGGGTGCAGTACATCAGCATGGGGCAAATTCTTTCCATCCCGATGATTGTCGCGGGTGTGATCATGATGGTCTGGGCATATCGTCGCAGCCCACAGCAACACGTTTCC**TCTAGA**GACTACAAAGACC |
| Body_Ec-Arm_Ef | pAP23 | CTATGACATGATTAC**GAATTC**GCGAAATAACAAGAAATTGTGGTGACAGATGACCAGTAGCTATCTGCATTTTCCGGAGTTTGATTCAATTGCATTTCGCCTTTTCGGTATTCCCGTCTATTGGTACGGCCTGATGTATCTGGTGGGTTTCATTTTTGCAATGTGGCTGGCAACACGACGGGCGAATCGTCCGGGCAGCGGCTGGACCAAAAATGAAGTTGAAAACTTACTCTATGCGGGCTTCCTCGGCGTCTTCCTCGGGGGACGTATTGGTTATGTTCTGTTCTACCAATGGCAAGATTATGTAGACAATCCGATTGAAATTTTCTTTGTCTGGGACGGCGGCATGTCTTTCCACGGCGGCCTGATTGGCGTTATCGTGGTGATGATTATCTTCGCCCGCCGTACTAAACGTTCCTTCTTCCAGGTCTCTGATTTTATCGCACCACTCATTCCGTTTGGTCTTGGTGCCGGGCGTCTGGGCAACTTTATTAACGGTGAATTGTGGGGCCGCGTTGACCCGAACTTCCCGTTTGCCATGCTGTTCCCTGGCTCCCGTACAGAAGATATTTTGCTGCTGCAAACCAACCCGCAGTGGCAATCCATTTTCGACACTTACGGTGTGCTGCCGCGCCACCCATCACAGCTTTACGAGCTGCTGCTGGAAGGTGTGGTGCTGTTTATTATCCTCAACCTGTATATTCGTAAACCACGCCCAATGGGAGCTGTCTCAGGTTTGTTCCTGATTGGTTACGGCGCGTTTCGCATCATTGTTGAGTTTTTCCGCCAGCCCGACGCGCAGTTTACCGGTGCCTGGGTGCAGTACATCAGCATGGGGCAAATTCTTTCCATCCCGATGATTGTCGCGGGTGTGATCATGATGGTCTGGGCATATCGTCGCAGCCCACAGCAACACGTTTCC**TCTAGA**GACTACAAAGACC |

Gene fragments were inserted between EcoRI and XbaI sites (shown in bold) in plasmid SLP14 by Gibson cloning.

**Suppl. Table 5. Residues exchanged in swap proteins.**

| Ec_Head^Hp^ | Ec head: G154-P197 | Hp head: G165-P189 |
| --- | --- | --- |
| Ec_Head^Mtub^ | Ec head: G154-P197 | Mtub head: G155-P195 |
| Ec_Head^Sa^ | Ec head: G154-P197 | Sa head: G148-P179 |
| Ec_Head^Ef^ | Ec head: G154-P197 | Ef head: G145-P175 |
| Ec_Arms^Ef^ | Ec arms: P13-W25 + F82-R94 | Ef arms: S7-W19 + W73-F85 |
| Ec_Arms-Head^Ef^ | Ec arms-head: P13-W25 + F82-R94 + G154-P197 | Ef arms-head: S7-W19 + W73-F85 + G145-P175 |
| Ef_Head^Ec^ | Ef head: G145-P175 | Ec head: G154-P197 |
| Ef_Arms^Ec^ | Ef arms: S7-W19 + W73-F85 | Ec arms: P13-W25 + F82-R94 |
| Ef_Arms-Head^Ec^ | Ef arms-head: S7-W19 + W73-F85 + G145-P175 | Ec arms-head: P13-W25 + F82-R94 + G154-P197 |

See corresponding data presented in Fig. 5.

References

1. Datsenko KA, Wanner BL. 2000. One-step inactivation of chromosomal genes in *Escherichia coli* K-12 using PCR products. Proc Natl Acad Sci USA 97:6640-6645.

2. Diao J, Komura R, Sano T, Pantua H, Storek KM, Inaba H, Ogawa H, Noland CL, Peng Y, Gloor SL, Yan D, Kang J, Katakam AK, Volny M, Liu P, Nickerson NN, Sandoval W, Austin CD, Murray J, Rutherford ST, Reichelt M, Xu Y, Xu M, Yanagida H, Nishikawa J, Reid PC, Cunningham CN, Kapadia SB. 2021. Inhibition of Escherichia coli Lipoprotein Diacylglyceryl Transferase Is Insensitive to Resistance Caused by Deletion of Braun's Lipoprotein. J Bacteriol 203:e0014921.

3. Legood S, Seng D, Boneca IG, Buddelmeijer N. 2022. A Defect in Lipoprotein Modification by Lgt Leads to Abnormal Morphology and Cell Death in Escherichia coli That Is Independent of Major Lipoprotein Lpp. J Bacteriol 204:e0016422.

4. Binet R, Wandersman C. 1995. Protein secretion by hybrid bacterial ABC-transporters: specific functions of the membrane ATPase and the membrane fusion protein. EMBO J 14:2298-2306.

5. Pailler J, Aucher W, Pires M, Buddelmeijer N. 2012. Phosphatidylglycerol::prolipoprotein diacylglyceryl transferase (Lgt) of Escherichia coli has seven transmembrane segments, and its essential residues are embedded in the membrane. J Bacteriol 194:2142-51.

6. Buddelmeijer N, Beckwith J. 2004. A complex of the Escherichia coli cell division proteins FtsL, FtsB and FtsQ forms independently of its localization to the septal region. Mol Microbiol 52:1315-27.
